# Application of single wrist-wearable accelerometry for objective motor diary assessment in fluctuating Parkinson’s disease

**DOI:** 10.1038/s41746-023-00937-1

**Published:** 2023-10-17

**Authors:** Matthias Löhle, Jonathan Timpka, Alexander Bremer, Hamid Khodakarami, Florin Gandor, Malcom Horne, Georg Ebersbach, Per Odin, Alexander Storch

**Affiliations:** 1 Department of Neurology, University Medical Center Rostock, Rostock, Germany; 2 German Center for Neurodegenerative Diseases (DZNE) Rostock-Greifswald, Rostock, Germany; 3 Division of Neurology, Department of Clinical Sciences Lund, Lund University, Lund, Sweden; 4 Department of Neurology, Skåne University Hospital, Lund, Sweden; 5 Global Kinetics Pty Ltd, 31 Queen St., Melbourne, VIC Australia; 6 Movement Disorders Hospital, Beelitz-Heilstätten, Beelitz, Germany; 7 Department of Neurology, Otto-von-Guericke University, Magdeburg, Germany; 8 Bionics Institute, Melbourne, VIC Australia; 9 The Department of Medicine, The University of Melbourne, St Vincent’s Hospital, Fitzroy, VIC 3010 Australia

**Keywords:** Parkinson's disease, Parkinson's disease

## Abstract

Advanced Parkinson’s disease (PD) is characterized by motor fluctuations including unpredictable oscillations remarkably impairing quality of life. Effective management and development of novel therapies for these response fluctuations largely depend on clinical rating instruments such as the widely-used PD home diary, which are associated with biases and errors. Recent advancements in digital health technologies provide user-friendly wearables that can be tailored for continuous monitoring of motor fluctuations. Their criterion validity under real-world conditions using clinical examination as the gold standard remains to be determined. We prospectively examined this validity of a wearable accelerometer-based digital Parkinson’s Motor Diary (adPMD) using the Parkinson’s Kinetigraph (PKG^®^) in an alternative application by converting its continuous data into one of the three motor categories of the PD home diary (Off, On and Dyskinetic state). Sixty-three out of 91 eligible participants with fluctuating PD (46% men, average age 66) had predefined sufficient adPMD datasets (>70% of half-hour periods) from 2 consecutive days. 92% of per-protocol assessments were completed. adPMD monitoring of daily times in motor states showed moderate validity for Off and Dyskinetic state (ICC = 0.43–0.51), while inter-rating methods agreements on half-hour-level can be characterized as poor (median Cohen’s *κ* = 0.13–0.21). Individualization of adPMD thresholds for transferring accelerometer data into diary categories improved temporal agreements up to moderate level for Dyskinetic state detection (median Cohen’s *κ* = 0.25–0.41). Here we report that adPMD real-world-monitoring captures daily times in Off and Dyskinetic state in advanced PD with moderate validities, while temporal agreement of adPMD and clinical observer diary data is limited.

## Introduction

Advanced stages of Parkinson’s disease (PD) are characterized by the presence of motor complications, which affect about 50% of patients after 5 and 90% after 10 years of disease^1–3^. During the disease course, most patients initially experience progressive shortening of benefit from short-acting dopaminergic drugs such as levodopa^3^, referred to as “Wearing-off”. The clinical assessment and classification of these transitions between the levodopa response and “Off” state have been comprehensively reviewed^4^ and are commonly referred to as “fluctuations”. With disease progression, patients tend to present with unpredictable Off periods and dyskinesias^5,6^. Dyskinesias are involuntary movements of head, trunk or extremities that can interfere with patient activity and lower quality of life (QoL)^7^. Unpredictable motor fluctuations are an important aspect for disease management in late stage PD with particularly high impact on health-related quality of life^8,9^. The clinical phenomenology of unpredictable motor fluctuations is multifaceted and includes among others “Delayed On”, “Dose failure/No-On” and “Random On-Off”^6,10^. The underlying pathophysiological mechanisms are complex and comprise not only factors affecting the peripheral pharmacokinetics of levodopa but also central (non-dopaminergic) mechanisms (for details, please refer to refs. ^6,10^). However, there is still an intensive debate on their precise phenomenology as well as their response to medication and the resulting treatment strategies^6,11^. Motor fluctuations are accompanied by fluctuations of non-motor symptoms, such as depression, anxiety and pain^12,13^. Since symptom fluctuations significantly impairs QoL, their accurate detection and subsequent treatment provide the opportunity to improve QoL in advanced PD^12,14^.

Clinical management and drug development for response fluctuations largely depend on clinical rating instruments such as the PD home diary^15^, Unified PD rating scale (UPDRS)^16^ or modified Abnormal Involuntary Movement Scale (mAIMS)^17^. Although these assessments are the current reference standard, they have biases and errors, since patients forget to record their motor state, do not adequately recognize the respective motor state, or confuse motor and non-motor symptoms^18–21^. The PD home diary was developed to quantify motor fluctuations. It is frequently used in clinical trials on motor fluctuations and fairly widespread in clinical routine^15,18,22^. For its use, patients are asked to indicate their predominant motor status during half-hour time periods throughout the day using the categories Asleep, Off state, On without dyskinesias (On state), and On with dyskinesia (Dyskinetic state). Recent validation studies however demonstrated that the PD home diary unsatisfactorily reflects actual motor states as compared to simultaneous clinical observation^19,23,24^. Consequently, there is a strong need for continuous and objective monitoring of motor function in PD.

Recent advancements in digital health technologies (DHTs) provide user-friendly wearables including smartwatch-based applications using inertial measurement unit sensors (e.g. accelerometers, gyroscopes) combined with sufficient battery life that can be tailored for continuous monitoring of motor symptom severity and thus presumably motor fluctuations^25,26^. Commercially available wearable devices such as STAT-ON (Sense4Care, Barcelona, Spain)^27,28^, Parkinson KinetiGraph^®^ (PKG^®^; Global Kinetics Corporation Ltd., Melbourne, Australia)^29–34^, and smartwatch applications such as StrivePD (Rune Labs Inc., San Francisco, USA), and Verily smartwatch (Verily Life Science, San Francisco, CA)^35^ have various characteristics (e.g. single versus multiple sensors) and deliverables to assess motor symptoms and their fluctuations in PD, but their application in clinical trials as well as their implementation in routine care is still limited^36,37^. However, the feasibility of using DHTs for measuring motor function/fluctuations has been demonstrated and first clinical evidence is provided that continuous, objective monitoring of motor symptoms using wearable biosensors may enhance clinical decision-making and outcome in PD patients^34,38^.

Continuous monitoring of motor symptoms in PD in an uncontrolled real-world environment without interrupting daily activities can be confounded by voluntary inactivity and (over)-activity (e.g. exercise) as well as tremor. Most DHTs overcome this problem by increasing the sample size by recording for several days and by reducing times of inactivity (evening) combined with subsequent averaging/smoothing^29,35,39,40^. It therefore remains largely unknown whether DHT data reflect the motor state at a given time point as an important information to manage unpredictable motor fluctuations and day-to-day variations in motor function. Available studies compared DHT outcomes with PD home diary data and demonstrated fair to good validity for daily times in the various motor states (mostly expressed as percentage daily times, PDT), but limited temporal agreement at a given time point (accuracy: 53%)^27,31^. Validity data of DHTs to continuously and temporarily monitor motor states similar to the PD home diary in an uncontrolled environment with clinical assessment as the outside criterion are not available.

**Fig. 1:**
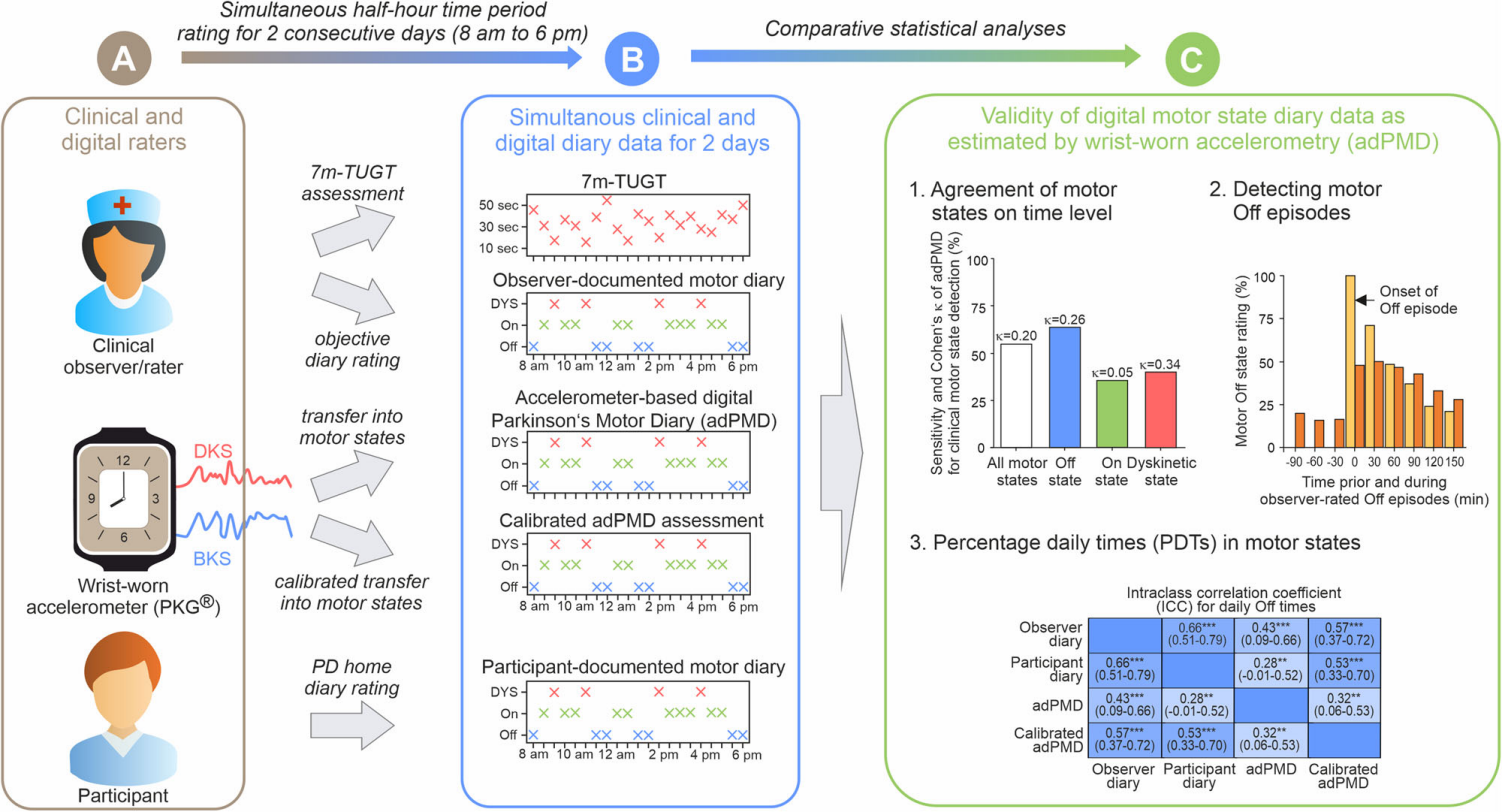
Graphical synopsis of application study of wrist-wearable accelerometry for objective motor diary assessment in fluctuating Parkinson’s disease. The present study was designed to validate wrist-wearable accelerometry-based digital Parkinson’s Motor Diary (adPMD) using the Parkinson’s Kinetigraph (PKG^®^) as the hardware device for objective motor diary assessment as an alternative application of this digital health technology (DHT). **A** Motor function was simultaneously assessed by fluctuating Parkinson’s disease (PD) patients using the PD home diary data of motor states (Off state, On state dyskinetic state) by the adPMD as well as by clinical observers and participants for each half-hour time period from 8 a.m. until 6 p.m. on 2 consecutive days. In addition, the 7 meter Timed-Up-and-Go test (7 m-TUGT) was performed simultaneously for each time period. **B** The quantitative accelerometer scores for bradykinesia (BKS) and dyskinesia (DKS) were transferred via median scores for each half-hour time period into motor state data similar to the diaries (Off state, On state and Dyskinetic state). **C** Comparative statistical analyses using the clinical rater diary data as the gold-standard outside criterion were applied to estimate the validity of adPMD as a clinically used wrist-worn accelerometry in PD for (1) temporal agreement of motor state ratings for the half-hour time periods, (2) detection of Off episodes following an On phase and (3) assessment of percentage daily times in the various motor states. Ancillary analyses used the PD home diary data as documented by study participants as an alternative outside criterion.

We here report on the VALIDATE-PD study^23,24^, which was designed to apply wrist-wearable accelerometry for objective motor diary assessment in fluctuating PD in a routine clinical environment (see Fig. 1 for study synopsis). Our main aim is to establish whether a wearable accelerometer-based digital Parkinson’s Motor Diary (adPMD) using the Parkinson’s Kinetigraph (PKG^®^) device can detect the clinical state at a point in time using simultaneous half-hourly performed clinical examinations by experienced raters with the PD home diary as the main comparator. Importantly, the PKG^®^ system was originally designed to use averaging of daily traces to aid routine clinical care and has no commercially available format of providing either two minutes or half-hour data nor have there ever been any claims for it to function in this time scale. We here use the PKG^®^ accelerometer system in an alternative manner and format by converting its continuous data into one of the three motor categories of the PD home diary resulting in adPMD ratings^15,22,31,41^. We then estimate the validity of the adPMD data with respect to three different diary outcomes: (1) Detecting the diary motor states at a given time point as routinely used clinical parameters to detect unpredictable Off state and Dyskinetic state periods. (2) Detecting Off episodes (Off state episode following motor On state) as another important aspect in routine care for adaption of medication^42^. (3) PDT of the motor states as a frequently used read-out in clinical trials^18,43–48^.

## Results

### Demographic and clinical data

In three centers in Germany and Sweden, we screened 96 participants for eligibility of whom 91 (95%) were successfully included into the VALIDATE-PD study according to inclusion and exclusion criteria. 63 participants (69%) had 2 days of calibrated adPMD datasets and were included in the final study analyses (see Participants & Methods section). Demographic and clinical characteristics of study cohort are displayed in Table 1. We did not observe relevant differences between the cohorts from the two countries. Clinical scores and antiparkinsonian medication were representative for an advanced PD cohort. A glossary of adPMD terms and clinical fluctuation parameters are displayed in Table 2.

**Table 1:** Demographic and clinical characteristics of adPMD study cohorts.

	Total cohort (*n* = 63)	German subcohort (*n* = 40)	Swedish subcohort (*n* = 23)	*P* value (cohorts)
Male/Female, *n* (%)	29 (46%)/34 (54%)	19 (48%)/21 (52%)	10 (44%)/13 (56%)	0.758^§^
Age, Median (IQR) in years	66 (60–73)	64 (57–69)	70 (60–76)	0.038^$^
Disease duration, Median (IQR) in years	9 (6–14)	10 (8–14)	7 (5–14)	0.159^$^
Symptom duration, Median (IQR) in years	11 (8–15)	11 (9–16)	8 (7–14)	0.076^$^
Duration of fluctuations, Median (IQR) in months	54 (34–99)	70 (38–106)	60 (33–86)	0.607^$^
Hypokinetic fluctuations	60 (35–103)	70 (38–106)	48 (20–86)	0.068^$^
Hyperkinetic fluctuations	36 (24–57)	39 (25–60)	35 (20–64)	0.453^$^
Clinical Phenotype				0.614^§^
Tremor Dominant, *n* (%)	11 (18%)	7 (18%)	4 (18%)	
Axial Dominant, *n* (%)	0 (0%)	0 (0%)	0 (0%)	
Appendicular Dominant, *n* (%)	7 (11%)	3 (8%)	4 (18%)	
Rigor Dominant, *n* (%)	1 (2%)	1 (3%)	0 (0%)	
Postural Instability and Gait Difficulty, *n* (%)	44 (70%)	30 (75%)	14 (64%)	
Reported motor complications during structured interview				
Nighttime off, *n* (%)	54 (86%)	34 (85%)	20 (87%)	1.000^§^
Wearing-off, *n* (%)	56 (89%)	37 (93%)	19 (83%)	0.247^§^
Delayed on, *n* (%)	35 (56%)	31 (78%)	4 (17%)	<0.001^§^
On-off phenomenon, *n* (%)	39 (62%)	26 (65%)	13 (59%)	0.645^§^
Peak-dose dyskinesia, *n* (%)	47 (75%)	31 (78%)	16 (70%)	0.486^§^
Biphasic dyskinesia, *n* (%)	12 (19%)	7 (18%)	5 (23%)	0.740^§^
Off-dose dystonia, *n* (%)	32 (51%)	22 (55%)	10 (48%)	0.583^§^
PD medications				
Levodopa, *n* (%)	63 (100%)	40 (100%)	23 (100%)	-
Dopamine agonizts, *n* (%)	43 (68%)	24 (60%)	19 (83%)	0.063^§^
MAO B inhibitors, *n* (%)	43 (68%)	28 (70%)	15 (65%)	0.695^§^
COMT inhibitors, *n* (%)	50 (79%)	36 (90%)	14 (61%)	0.009^§^
Amantadine, *n* (%)	25 (40%)	20 (50%)	5 (22%)	0.027^§^
Levodopa dose (mg per day), Median (IQR)	550 (450–725)	506 (425–700)	550 (500–875)	0.054^$^
Total levodopa equivalent dose (mg per day), Median (IQR), calculated according to^68^	1199 (976–1505)	1331 (1030–1635)	1112 (814–1263)	0.003^$^
Clinical scales				
MDS-PDRS Total score On state, Median (IQR)	63 (45–82)	64 (52–86)	53 (39–58)	0.007^$^
Part I	11 (8–16)	13 (8–14)	13 (7–16)	0.133^$^
Part II	14 (9–19)	16 (10–21)	11 (7–17)	0.039^$^
Part III	28 (18–40)	28 (21–41)	25 (16–31)	0.149^$^
Part IV	8 (6–10)	9 (7–10)	6 (4–8)	0.001^$^
Hoehn & Yahr stage, Median (IQR)	4 (3–5)	4 (3–4)	4 (3–5)	0.676^+^
Montreal Cognative Assessment score, Median (IQR)	27 (25–28)	27 (26–28)	26 (24–28)	0.111^$^
Beck’sches Depression Inventory score, Median (IQR), assessed in German cohort only	10 (4–16)	10 (4–16)		

^§^*P* values are from χ^2^ test or fisher exact test as appropriate.

^$^*P* values are from Mann-Withney *U* test.

^+^*P* values from Jonckheere Terpstra test.

**Table 2:** Glossary of PKG^®^ and wrist-wearable accelerometer-based digital Parkinson’s Motor Diary (adPMD) terms and clinical parameters.

Parameter	Abbreviation	Explanation	Ref.
PKG® quantitative scores per time periods			
Median bradykinesia score	mBKS	30 min median of bradykinesia score <80 (inactivity/non-wrist wear excluded) for diary time period	^29^
Timed bradykinesia score	mBKS_timed (28)_	28 min median bradykinesia score with inactivity/non-wrist wear excluded from recordings exactly 28 min around clinical testing (seven 2-min epochs prior and seven 2-min-epochs after clinical testing), German cohort only	
Timed bradykinesia score	mBKS_timed (16)_	16 min median bradykinesia score with inactivity/non-wrist wear excluded from recordings exactly 16 min (eight 2-min epochs) prior to but excluding clinical testing, German cohort only	
Dyskinesia score	mDKS	30 min median of dyskinesia score with activity excluded for diary time period	^29^
Timed dyskinesia score	mDKS_timed (28)_	28 min median dyskinesia score with activity excluded from recordings exactly 28 min around clinical testing (seven 2-min epochs prior and seven 2-min epochs after clinical testing), German cohort only	
Wrist-wearable accelerometer-based digital Parkinson’s motor diary (uncalibrated/calibrated adPMD) data per half-hour periods			
Asleep	-	Asleep as predicted by adPMD through thresholding of BKS (uncalibrated data) or by adPMD through per patient calibration of threshold of 30 min median BKS using day 0 of patient diary (calibrated data)	^31^
Off state	Off state	Motor Off state as predicted by adPMD through thresholding of 30 min median BKS (uncalibrated data) or by adPMD through per patient calibration of threshold of 30 min median bradykinesia scores using day 0 of patient diary (calibrated data)	^31^
On without dyskinesia state	On state	Motor On without dyskinesia state as predicted by adPMD through thresholding of 30 min median BKS and DKS (uncalibrated data) or by adPMD through per patient calibration of threshold of 30 min median BKS and DKS using day 0 of patient diary (calibrated)	^31^
On with dyskinesia state	Dyskinetic state	Motor On with dyskinesia state as predicted by adPMD through thresholding of 30 min median DKS (uncalibrated data) or by adPMD through per patient calibration of threshold of 30 min median dyskinesia score using day 0 of patient diary (calibrated data)	^31^
Observer or participant diary data per half-hour periods			
Asleep	-	Asleep as rated by clinical observer (experienced movement disorder specialist) or study participant	^24^
Off state	Off state	Motor Off state as rated by clinical observer (experienced movement disorder specialist) or study participant	^24^
On without dyskinesia state	On state	Motor On without dyskinesia state as rated by clinical observer (experienced movement disorder specialist) or study participant	^24^
On with dyskinesia state	Dyskinetic state	Motor On with dyskinesia state as rated by clinical observer (experienced movement disorder specialist) or study participant	^24^
Off episode	Off episode	Off state episode of at least 0.5 h duration following a motor On state period of at least 1.5 h	^42^
Off time	Off time	Daily time in Off state (between 8 a.m. and 6 p.m.), expressed in percentage of total half-hours with diary data (PDT)	
On without dyskinesia time	On time	Daily time in On state (between 8 a.m. and 6 p.m.), expressed in percentage of total half-hours with diary data (PDT)	
On with dyskinesia time	Dyskinetic time	Daily time in Dyskinetic state (between 8 a.m. and 6 p.m.), expressed in percentage of total half-hours with diary data (PDT)	
Other clinical parameters			
7 m-Timed Up and Go Test	7m-TUGT	Modified 7 m-TUGT test results as a quantitative clinical measure of bradykinesia	^58^

### Temporal agreement of adPMD motor state classification with clinical diary data

To allow for direct comparisons of adPMD and frequent clinical motor assessment data, quantitative PKG^®^ scores were transferred into motor states as decribed previously^31^ with the routinely used consistent threshold (75^th^ percentile) in 30 min time periods resulting in adPMD ratings. In total, 2384 (90.1% of all periods or 97.5% of all adPMD scorings) half-hour PKG^®^ quantitative scores were classifiable into motor states with 6.0% classified as Asleep, 40.9% as motor Off state, 32.7% as On state and 20.4% as Dyskinetic state.

**Fig. 2:**
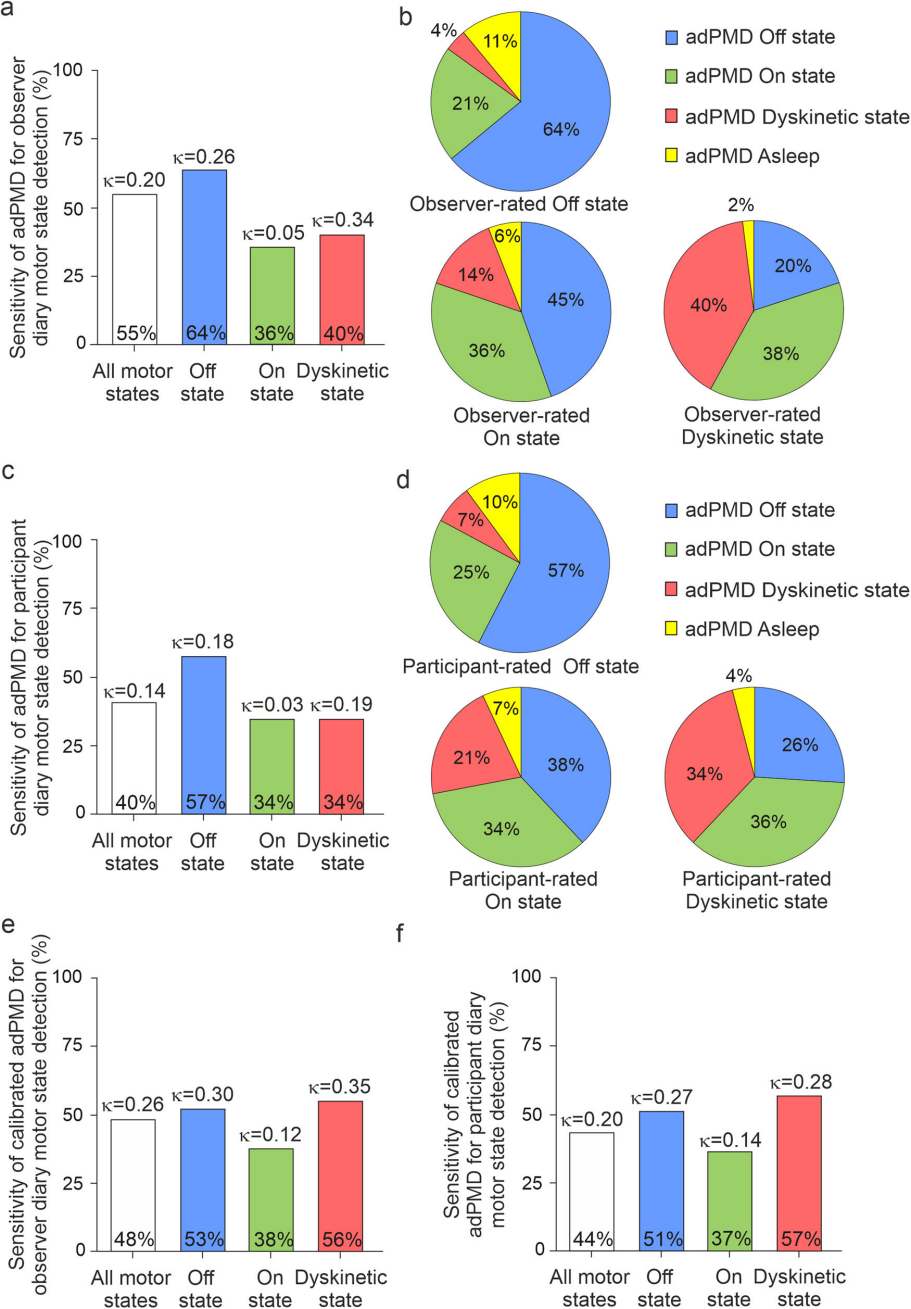
Temporal agreement of uncalibrated and calibrated wearable accelerometer-based digital Parkinson’s Motor Diary (adPMD) ratings and observer- and participant-documented diary motor states on the half-hour time period level. **a**, **c** Sensitivities expressed in percent and Cohen’s κ values of uncalibrated adPMD ratings for the detection all motor states (white color), Off state (blue color), On state (green color) and Dyskinetic state (red color) as rated by clinical observers (**a**) and by participants (**c**) using the PD Home diary categories. Preferred choices in observer-documented diaries (**b**) and participant diaries (**d**) in the respective adPMD motor states. Figures are based on 2339 (88.4% of all periods) simultaneous half-hourly adPMD and clinical observer ratings (**a**, **b**) and 2338 (88.4% of all time periods) simultaneous adPMD and participant ratings of 63 participants (**c**, **d**). Sensitivities in percent and Cohen’s κ values of calibrated adPMD ratings for the detection all motor states, Off state, On state and Dyskinetic state as rated by clinical observers (**e**) and participants (**f**) using the PD home diary (1877 [70.9%] and 1913 [72.3%] half-hour periods with simultaneous observer or participant ratings of 63 participants; for preferred choices, refer to Supplementary Figs. 4a, b). For additional test performance measures, refer to Table 3, Supplementary Tables 1 and 2.

To compare adPMD data with clinical observer motor diary data, we next estimated the sensitivities of adPMD for the detection of respective motor states as rated by clinical observers on the group level (Fig. 2a): adPMD ratings correctly recognized 55% of all motor states, but 64% of observed Off states, only 36% of On and 40% of Dyskinetic state simultaneous to the observer. Together with the specificities of adPMD ratings, these figures translated into balanced accuracies (avarage between sensitivy and specificity) between 52% for On state and 65% for Off and Dyskinetic state (Table 3, Supplementary Table 1). Corresponding Cohen’s *κ* values ranged from 0.05 for On state to 0.34 for Dyskinetic state. The adPMD rated On state in 21% of the intervals with observed Off state (Fig. 2b). Even more strikingly, the adPMD decided Off in 45% of those intervals in which the observer had actually noted On state.

**Table 3:** Validity parameters of the adPMD according to the various cut-offs as determined by ROC plot analyses for transferring PKG^®^ scores into adPMD states.

	ROC-AUC (95%CI)	adPMD test validity measures on group level				adPMD test validity measures on participant level, median (95%CI)				Percentage daily times, median (95%CI)
		Sensitivity, recall (%)	Specificity (%)	Balanced accuracy (%)	Cohen’s *κ*	Sensitivity, recall (%)	Specificity (%)	Balanced accuracy (%)	Cohen’s *κ*	ICC
Discrimination of Off versus Non-Off state from simultaneous clinical observer ratings										
mBKS (standard cut-off)	0.740 (0.718–0.762)^***^	64%	67%	65%	0.26	58% (0–100%)	67% (11–100%)	58% (39–83%)	0.13 (−0.15–0.61)	0.43 (0.09–0.66)^+++^
mBKS (calibrated cut-off)	0.867 (0.854–0.880)^***^	53%	79%	66%	0.30	47% (0–100%)	83% (31–100%)^##^	56% (43–83%)	0.20 (−0.16–0.64)	0.57 (0.37–0.72)^+++^
mBKS (individualized cut-offs, all participants)^a^	0.715 (0.691–0.738)^***^	62%	74%	68%	0.31	67% (25–100%)	72% (35–100%)	67% (41–90%)^##,§§^	0.25 (−0.01–0.68)^##,§^	0.57 (0.38–0.82)^+++^
mBKS (individualized cut-offs, sig. ROC only)^a,b^	0.822 (0.791–0.853)^***^	70%	80%	75%	0.48	77% (34–100%)	80% (58–100%)	81% (59–88%)	0.45 (0.23–0.75)	0.74 (0.47–0.89)^+++^
Discrimination of Dyskinetic state versus Non-dyskinetic state from simultaneous clinical observer ratings										
mDKS (standard cut-off)	0.774 (0.754–0.793)^***^	40%	90%	65%	0.34	26% (0–88%)	95% (31–100%)	55% (45–83%)	0.21 (−0.13–0.79)	0.51 (0.18–0.71)^+++^
mDKS (calibrated cut-off)	0.842 (0.828–0.857)^***^	56%	78%	67%	0.35	57% (0–100%)^#^	84% (0–100%)^###^	58% (46–92%)	0.35 (−0.05–0.83)	0.20 (−0.05–0.42)
mDKS (individualized cut-offs, all participants)^a^	0.748 (0.727–0.770)^***^	75%	69%	72%	0.41	75% (30–100%)^##,§^	75% (25–100%)^###^	71% (49–94%)^###,§§§^	0.41 (−0.03–0.88)^#,§^	0.34 (0.04–0.59)+
mDKS (individualized cut-offs, sig. ROC only)^a,b^	0.811 (0.785–0.837)^***^	74%	83%	79%	0.57	81% (46–100%)	81% (67–100%)	80% (68–96%)	0.54 (0.27–0.93)	0.49 (0.10–0.76)^+^

^***^Represents *P* < 0.001 from ROC-AUC calculation.

^+^Represents *P* < 0.05 and ^+++^*P* < 0.001 from intraclass correlation coefficient (ICC) calculation.

^#^Represents *P* < 0.05, ^##^*P* < 0.01, ^###^*P* < 0.001 when compared to respective result with adPMD standard cut-off, ^§^*P* < 0.05, ^§§^*P* < 0.01, ^§§§^*P* < 0.001 when compared to results with calibrated adPMD cut-offs from Friedman tests with post-hoc Wilcoxon test with Bonferroni adjustment for α inflation.

^a^Individual cut-offs were estimated by ROC analysis for each participant for the discrimination between observer Off state and Non-Off state by mBKS and between observer-documented Dyskinetic and Non-Dyskinetic state by mDKS.

^b^Only participants with significant discrimination in ROC analyses (AUC significantly larger than 0.5) between observer Off state and Non-Off state by mBKS (*n* = 20) and between observer-documented Dyskinetic and Non-Dyskinetic state by mDKS (*n* = 29) were used for these analyses (not included in comparative statistics).

In ancillary analyses, we calculated various validity measures of the adPMD ratings for the detection of diary motor state data from study participants (PD Home diary data). The adPMD correctly recognized 40% of all motor states, but 57% of patient-rated Off states, 34% of On states, and 34% of Dyskinetic state simultaneous to the participant’s rating in their PD home diaries (Fig. 2c, see Supplementary Table 2 for other validity measures). The adPMD rated On state in 25% of the intervals with participant-rated Off state (Fig. 2d). Moreover, the adPMD decided Off or On state in 38% and 34%, respectively, of those intervals in which the participants had actually noted On state.

Comparisons of true ratings (true positives+true negatives) by adPMD between clinical observer and participant diary data revealed significantly more true ratings of adPMD for detecting observer as compared to participant ratings when analyzing all motor states or only Off state (*P* < 0.001; McNemar test), but no relevant differences for On and Dyskinetic states (*P* ≥ 0.05; McNemar tests). Similar results were obtained for true positive ratings (sensitivity) and true negative ratings (specificity; data not shown).

To address potential patient-inherent factors, we calibrated adPMD data to model the first day of diary entries by each participant (PD Home diary data) for Off and Dyskinetic state. We thus used algorithms classifying the continuous accelerometer data into motor states for every half-hour time period between 8 a.m. and 6 p.m. using scores and diary ratings from day 0 as decribed earlier^31^. Test performance measures including balanced accuracies and Cohen’s *κ* values were similar to those estimated by using uncalibrated adPMD data (Fig. 2e, f; Table 3; Supplementary Fig. 1, Supplementary Tables 1,2).

### Validation of adPMD classifications on participant level

**Fig. 3:**
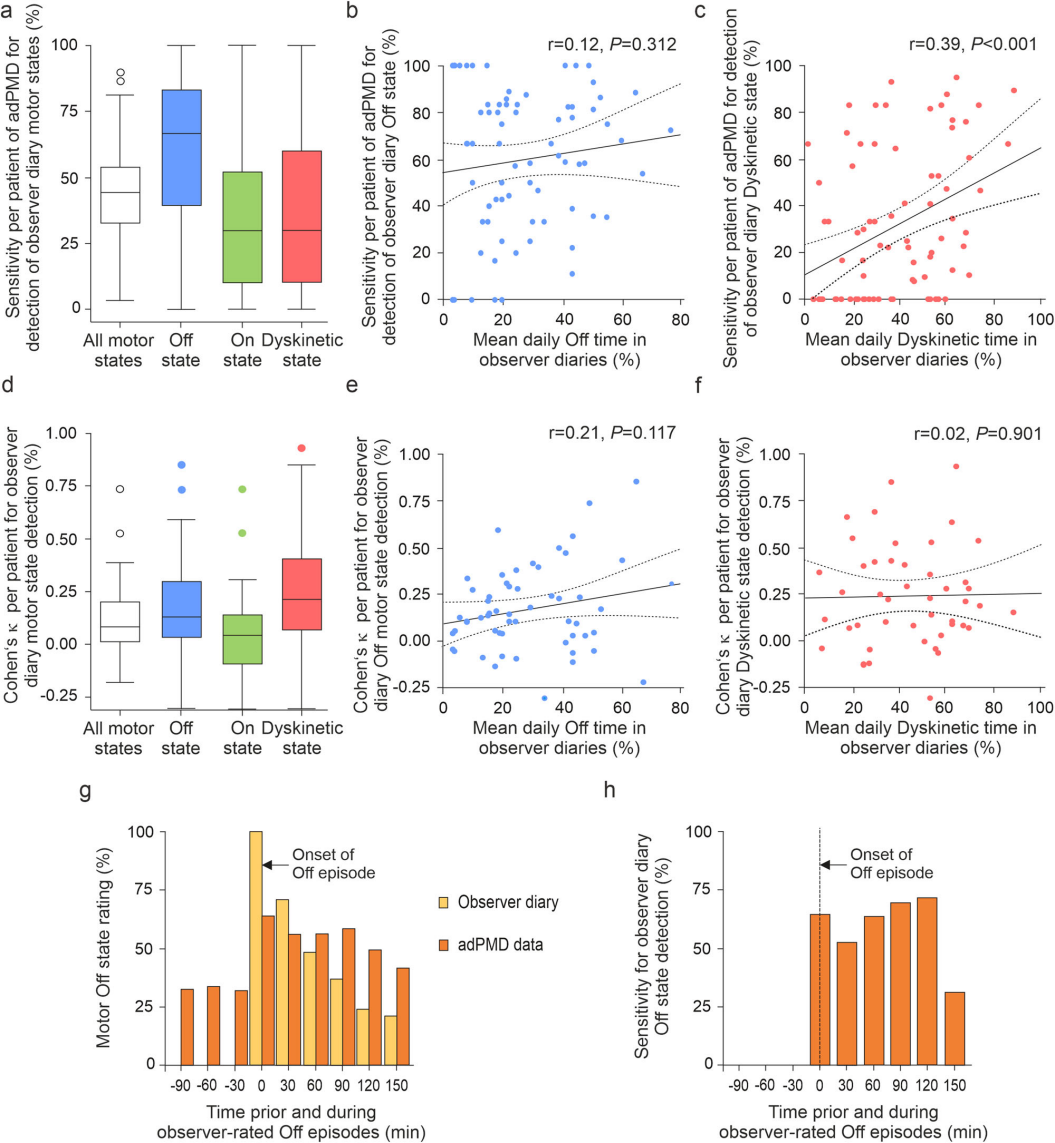
Temporal agreement between wearable accelerometer-based digital Parkinson’s Motor Diary (adPMD) ratings and observer-documented motor states on the participant level and during the time course of Off episodes. Sensitivities (**a**–**c**) and Cohen’s *κ* values (**d**–**f**) of uncalibrated adPMD data for the detection observer-documented motor states on the participant level. Sensitivities expressed in percent (**a**) and Cohen’s *κ* values (**d**) for all motor states (white color), Off state (blue color), On state (green color) and Dyskinetic state (red color) based on simultaneous half-hourly adPMD and clinical observer ratings in 63 participants. Boxplots are shown with a central mark at the median, bottom, and top edges of the boxes at 25^th^ and 75^th^ percentiles, respectively, whiskers out to the most extreme points within 1.5 times the interquartile range, and outliers scoring more than 1.5 × IQR but at most 3 × IQR outside the quartiles. Correlation analyses of sensitivities and percentage daily times for Off state (**b**) and Dyskinetic state (**c**). Correlation analyses of Cohen’s *κ* values and percentage daily times for Off state (**e**) and Dyskinetic state (**f**). Numbers in right corner of diagrams (**b**, **c**, **e**, **f**) represent Pearson correlation coefficients and *P* values. For additional test performance measures, refer to Table 3. **g**, **h** Temporal agreement between adPMD and observer-documented motor states during the time course of Off episodes. Off episodes were defined as a minimum of 30 min Off preceded by at least 90 min with On time as judged by the clinical observers. **g** Proportions of Off responses on simultaneous observer ratings (yellow color) and adPMD motor states (brown color) synchronized to the onset of observer-rated Off episodes. **h** Sensitivities of uncalibrated adPMD ratings for the detection of observer Off ratings synchronized to the onset of the observer-documented Off episodes. Values are provided as numbers from 84 Off episodes in 51 participants.

Validity of adPMD data when compared to observer diary data on the participant level were estimated using the sensitivity, specifitity, balanced accuracy and Cohen’s *κ* for each participant (Fig. 3, Table 3). Taking the observer diary as gold standard criterion, median sensitivities of adPMD data were 41% of all observer-rated motor states, 67% of Off states, but only 27% of On state and 23% of Dyskinetic state (Fig. 3a). The corresponding median balanced accuracies ranged from 52% to 58% and Cohen’s *κ* values from 0.04 to 0.21 (Fig. 3b, Table 3). Importantly, only the sensitivities for Dyskinetic state showed a moderate correlation with the respective PDTs (“number of positive hits”), but all other validity measures—particularely all Cohen’s *κ* values—did not correlate with the magnitude of test outcomes (PDTs) making a relevant proportional bias unlikely (*P* ≥ 0.05 from Pearson’s correlation test; Fig. 3b, c, e, f). In addition, the validity measures were not associated with potential confounding demographic or clinical parameters (*P* ≥ 0.05 from Mann-Whitney *U* test or Pearson’s correlation test as appropriate; see Table 1 for covariable list, data not shown). Calibration of adPMD data did not relevantly change validity measures of adPMD for the detection of clinical observer diary motor states (see Supplementary Fig. 2a, b. Table 3). Similar results were obtained for the test performance measures including sensitivities and Cohen’s *κ* for each participant of adPMD data with participant-rated diary data (Supplementary Fig. 2c–f).

### Agreement of adPMD classification with clinical ratings during Off episodes

Rather than compare adPMD and diary scores from half-hour periods, the timing of adPMD scores was synchronized with Off episodes^42^. Off episodes were defined as an Off state period of at least 0.5 h duration following a motor On state period of at least 1.5 h (Supplementary Fig. 3). We did not observe relevant changes of median bradykinesia scores (mBKS) at the beginning of the Off episodes with an increase of 12% (IQR: −9 to 53%) for observer-reported Off episodes (*P* = 0.615; Mann-Whitney *U* test). Consistently, there was no change in 7m-TUGT as a quantitative clinical bradykinesia outcome measure (17% [8–35%]; *P* = 0.693; Mann-Whitney U-test). There was, however, a moderate correlation between the change in mBKS with the change of 7m-TUGT results at the start of observer Off episodes (*r* = 0.364, *P* = 0.007; Pearson’s correlation test).

We next analyzed the temporal agreement of adPMD ratings and clinical diary ratings with respect to the time course of Off episodes (Fig. 3g, h). Sensitivities during the first 2.5 h of the Off episodes ranged from 31% to 72% for adPMD data with no major differences over the time course of Off episodes. Corresponding balanced accuracy values ranged from 56% to 79% and Cohen’s *κ* values from −0.15 to 0.18. Calibration of adPMD data to participants’ individual thresholds did not lead to relevant differences with sensitivities of 43–60%, balanced accuracies of 59–78%, and Cohen’s *κ* values of −0.22 to 0.25 (Supplementary Fig. 4; for data on participant-documented diaries, refer to Supplementary Results).

### Correlation of PDTs between adPMD data and clinical ratings

Daily times spent in the three different motor states (expressed here as percentage daily times; PDTs) calculated from PD home diary have been repeatedly used as the primary outcome measures in clinical trials on motor fluctuations in advanced PD^18^.

**Fig. 4:**
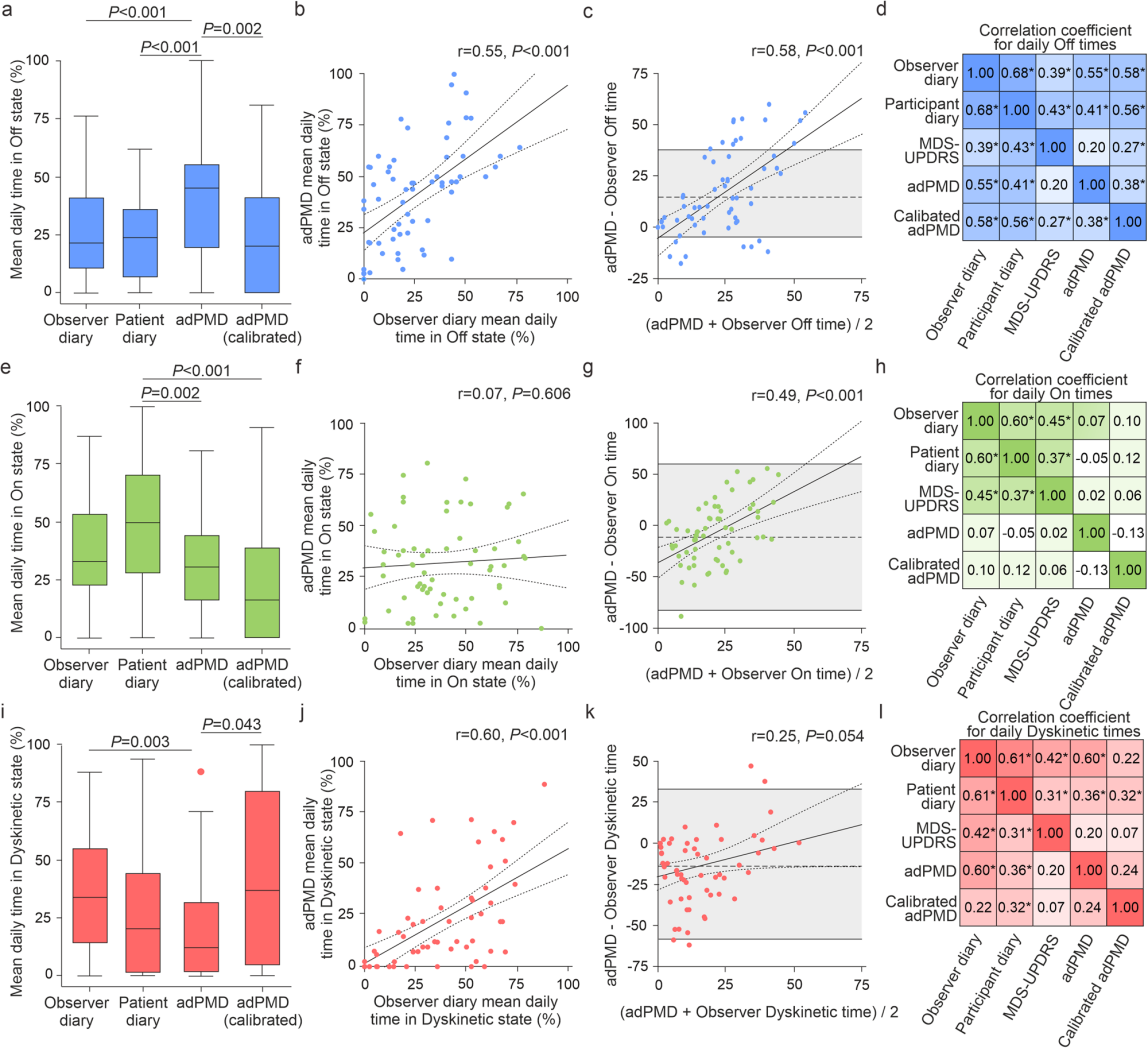
Proportions for times spent of motor states as assessed by observer diaries, participant diaries and wearable accelerometer-based digital Parkinson’s Motor Diary (adPMD) ratings. Distribution of daily Off times (**a**), On times (**e**) and Dyskinetic times (**i**) based on the simultaneous, half-hourly performed clinical diary/adPMD ratings from 63 participants from 2 consecutive days (8 am to 6 pm). Boxplots are shown with a central mark at the median, bottom, and top edges of the boxes at 25^th^ and 75^th^ percentiles, respectively, whiskers out to the most extreme points within 1.5 times the interquartile range, and outliers scoring more than 1.5 × IQR but at most 3 × IQR outside the quartiles. Displayed *P* values are from from Friedman tests with *post-hoc* Wilcoxon signed-rank tests with Bonferroni correction for multiple comparisons. Correlation analyses of daily Off times displaying exemplarily the scatter plot of calibrated adPMD and observer Off times and the corresponding Bland-Altman plot (**b**, **c**) and the corellation matrix (**d**). Correlation analyses of daily On times displaying exemplarily the scatter plot of calibrated adPMD and observer On times and the corresponding Bland-Altman plot (**f**, **g**) and the correlation matrix (**h**). **j**–**l** Correlation analyses of daily Dyskinetic times displaying exemplarily the scatter plot of calibrated adPMD and observer Dyskinetic times and the corresponding Bland-Altman plot (**b**, **c**) and the correlation matrix (**d**). Solid lines in scatter plots represent the regression line with 95%CI (dotted lines) and the gray area in (**c**, **g**, **k**) the limit of argreement (mean ± 1.96 SD). Numbers in right corner of scatter plots represent Pearson correlation coefficients and *P* values. In correlation matrices, * represent *P* < 0.05, ** *P* < 0.01 and *** *P* < 0.001 from Pearson’s correlation tests.

On the group level, we detected similar PDTs for motor On state when comparing adPMD and observer-rated diary data with medians of 33% and 31% daily On time for observer and adPMD data (Fig. 4), but different PDTs for Off and Dyskinetic state with medians of 21% and 46% Off time and 34% and 8% Dyskinetic time for observer and adPMD data. Calibration of adPMD data resulted in an increase in Dyskinetic time at cost of Off time leading to an overall better association of adPMD data with observer diary data on the group level (Fig. 4).

**Fig. 5:**
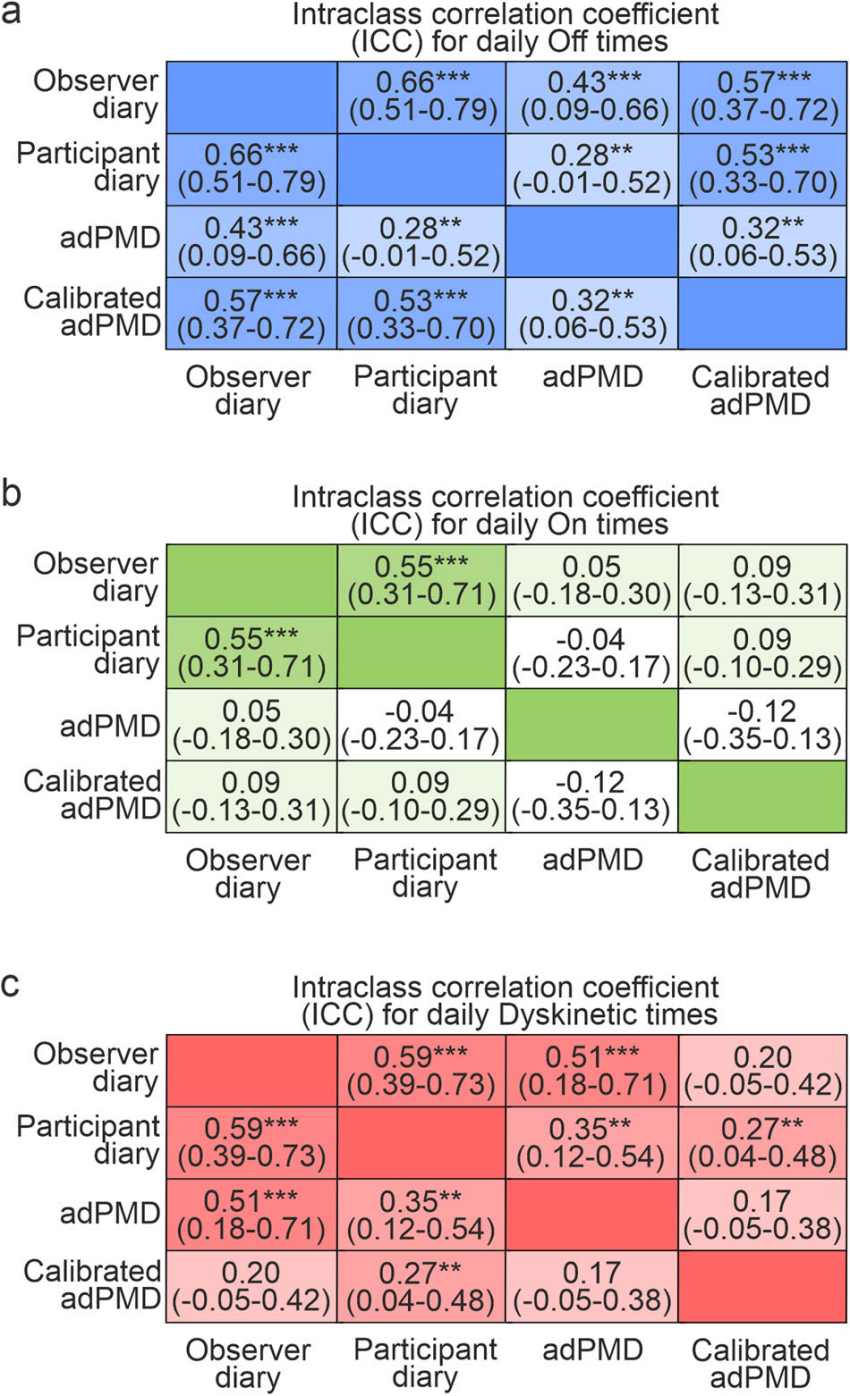
Reliability (intraclass correlation coeficient; ICC) of motor state times as assessed by observer diaries, participant diaries and wearable accelerometer-based digital Parkinson’s Motor Diary (adPMD) ratings. ICC values and their 95%CIs of proportions of Off times (**a**), On times (**b**) and Dyskinetic times (**c**). ICC estimates and their 95%CIs were calculated based on single-rating, absolute-agreement, 2-way mixed-effects models with two rating instruments across 63 participants. ** represents *P* < 0.01 and ***P < 0.001 from ICC analyses.

Analyses on the participant level using Pearson correlation analyses of individual PDTs revealed moderate correlations of Off time between adPMD and observer diary data with moderate proportional bias in Bland–Altman plot, but only a weak correlation with MDS-UPDRS percentage Off time (Fig. 4b–d). No relevant correlations of adPMD and clinical diary data were detected for On times (Fig. 4f–h). For Dyskinetic time, we detected a strong correlation between adPMD and observer diary data with no proportional bias, but only a weak correlation between calibrated adPMD data and clinical observer data (Fig. 4j–l). Multivariate regression analyses controlling for the candidate co-variates age, sex, symptom duration, fluctuation duration, disease severity (MDS-UPDRS III motor score), MDS-UPDRS part IV as a measure of motor complication, BDI and MoCA largely confirmed these correlation test results (Supplementary Table 3). Validity analyses using ICC calculation revealed moderate validity for adPMD ratings for Off time and Dyskinetic time when correlated with observer diary data (Fig. 5). Calibration of adPMD data did not lead to relevant changes for most measures, but did reduce the ICC value for Dyskinetic time.

### Factors that influence validity of adPMD motor state assessment

**Fig. 6:**
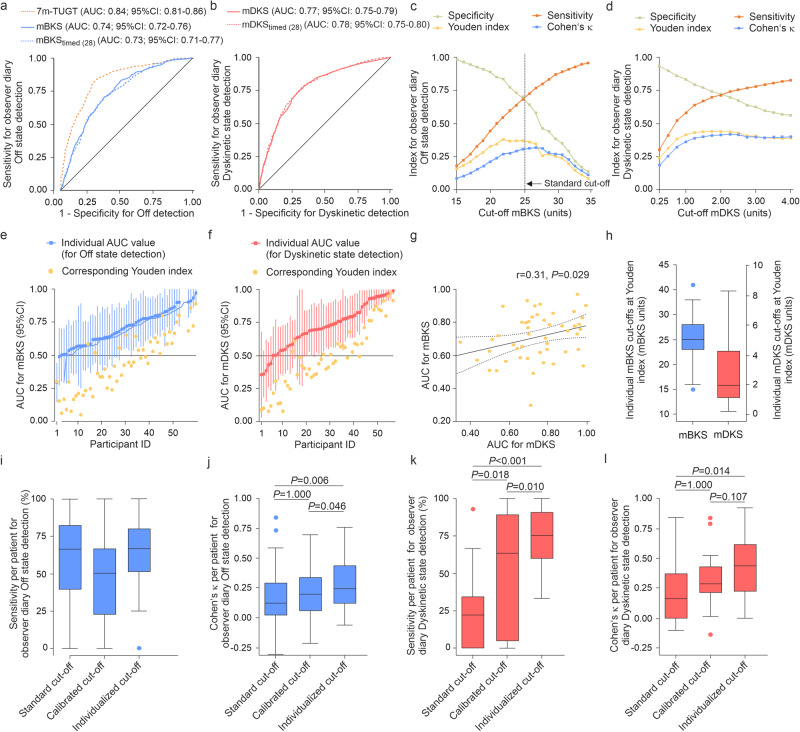
Test performance of PKG^®^ quatitative scores for the detection of observer-documented Off and Dyskinetic state. **a**–**c** Test performance of mBKS transferred into motor diary Off state. **a** Receiver operating curve (ROC) analyses displaying the sensitivity and specificity of the 7m-TUGT results, mBKS and exactly timed mBKS recordings for diary classification with time frames of the 28 min (seven 2-min epoches before and 7 epoches after clinical rating) around the clinical ratings (mBKS_timed (28)_) for the detection of observer-documented Off state and (**b**) the individual AUC values and Youden values of mBKS for the detection the detection of observer-documented Off state. **c** Test performance indices (sensitivity, specificity, Youden index, Cohen’s *κ* for the detection of observer-documented Off state by mBKS cut-offs. Note the standard cut-off value of 25 units used for clinical routine use. **d**–**f** Test performance of mDKS tranferred into motor diary Dyskinetic state. **d** ROC analyses displaying the sensitivity and specificity of mDKS and exactly timed mDKS recordings for diary classification with time frames of the 28 min (seven 2-minutes epoches before and 7 epoches after clinical rating) around the clinical ratings (mDKS_timed (28)_) for the detection of observer-documented Dyskinetic state and (**b**) the individual AUC values and Youden values of mDKS for the detection the detection of observer-documented Dyskinetic state. **c** Test performance indices (sensitivity, specificity. Youden index, Cohen’s *κ* for the detection of observer-documented Dyskinetic state by mDKS cut-offs. **g** Correlation of individual AUC values of mBKS and mDKS for the detection of the respective motor state. Numbers in right corner of scatter plot represent Pearson correlation coefficient and *P* value. **h** Distribution of individual mBKS and mDKS cut-offs values for detection of the respective motor state as rated by the clinical observer estimated by ROC analyses normalized to the 50th percentile of normal subjects (mBKS: 25 units; mDKS: 4 units). **i**–**l** Temporal agreement between wrist-wearable accelerometer-based digital Parkinson’s Motor Diary (adPMD) ratings ratings and observer-documented motor states on the participant level using the various cut-offs. Sensitivites expressed in precent (**i**, **k**) and Cohen’s *κ* values (**j**, **l**) of adPMD ratings for the detection of observer-documented Off state and Dyskinetic state on the participant level. Displayed *P*-values are from from Friedmann tests with post-hoc Wilcoxon signed-rank tests with Bonferroni correction for multiple comparisons. Boxplots (**h**–**l**) are shown with a central mark at the median, bottom, and top edges of the boxes at 25^th^ and 75^th^ percentiles, respectively, whiskers out to the most extreme points within 1.5 times the interquartile range, and outliers scoring more than 1.5 × IQR but at most 3 × IQR outside the quartiles.Data are based on 2389 simultaneous half-hourly performed observer diary ratings, respectively with simultaneous PKG^®^ recordings from 63 participants from 2 consecutive days (8 a.m. to 6 p.m.). 7m-TUGT and timed PKG^®^ recordings were only performed in the German cohort (*n* = 40, 1361 half-hourly assessments).

To determine factors influencing the transfer of continuous PKG^®^ quantitative scores into ordinal adPMD ratings and thus the validity of adPMD motor state assessment, we first determined the influence of thresholding on the validity of temporal agreement of adPMD ratings. Receiver operating curve (ROC) analyses revealed significant sensitivity-specificity relationships for both mBKS and 7m-TUGT results for detecting observer-documented Off state and median dyskinesia scores (mDKS) for the detection of Dyskinetic state (Fig. 6a–d, for precision-recall curve [PRC] plot analyses, refer to Supplementary Text, Supplementary Table 1). The optimal cut-off value (at maximal Youden index) for standard mBKS to discrimate Off versus Non-Off states was 25 units, leading to a maximal Cohen’s κ of 0.303. For Dyskinetic state detection, the optimal cut-off of standard mDKS scoring was 2 units with a maximal Cohen’s *κ* of 0.409.

We then determined the individual optimal cut-off values for mBKS and mDKS for the detection of the respective motor states for each participant by using ROC analyses. Discrimination (AUC significantly larger than 0.5) between observer Off state and Non-Off states by mBKS succeded in 20 out of 56 participants with Off state periods (36%), and between observer-documented Dyskinetic and Non-Dyskinetic states by mDKS in 29 out of 56 participants with Dyskinetic state periods (52%; *P* = 0.063; *χ*^2^ test; Fig. 6e–g, for PRC plot analyses, refer to Supplementary Text, Supplementary Fig. 5). By searching for potential factors predicting significant discrimination of Off and Dyskinetic state by the respective PKG^®^ scores, we did not detect any significant association of major demographic, clinical and observer diary parameters with individual thresholding data (see Supplementary Results, Supplementary Fig. 6a, b for details).

The ranges of individual cut-offs were wide for both mBKS and mDKS (Fig. 6h) with a median Youden index of 0.44 (95%CI: 0.07–0.81) for mBKS, and 0.49 (95%CI: 0.09–0.92) for mDKS. Using these individual cut-offs did—in general—not change the test performance for detecting Off state using mBKS or Dyskinetic state using mDKS (Table 3; Supplementary Fig. 6c, d). However, analyzing only individuals with relevant discrimination in ROC analyses led to a maximal Cohen’s *κ* of 0.484 for mBKS and Off detection and a maximal Cohen’s κ of 0.574 for mDKS and Dyskinetic state detection (Table 3; Supplementary Fig. 6e, f).

Using the individualized cut-offs, the adPMD test performance measures on the group level did not change for Off state detection as compared to standard adPMD cut-offs, but raised for Dyskinetic state detection with Cohen’s *κ* value indicating moderate validity (Table 3). On the participant level, the median balanced accuracy and Cohen’s *κ* for Off and Dyskinetic state were significanty increased, for the Dyskinetic state to the moderate validity level (Fig. 6i–l; Table 3). Selecting participants with significant ROC analyses results, test performance measures showed moderate validity for both Off and Dyskinetic state detection (Table 3).

Validity analyses of PDTs from adPMD ratings with individualized thresholds using ICC calculations revealed no relevant changes when compared to standard cut-off adPMD assessments with moderate validity for individualized adPMD data for Off times (ICC = 0.57 [95%CI: 0.38–0.82]) and Dyskinetic time estimation (ICC = 0.34 [95%CI: 0.04–0.59]; Table 3).

We then analyzed the importance of the timing of PKG^®^ recordings with respect to the clinical rating time for the validity of the temporal agreement and PDTs of the adPMD ratings. Using exactly timed PKG^®^ recordings for diary classification with time frames of the 28 min around the clinical ratings (mBKS/DKS_timed (28)_) or the 16 min directly prior clinical rating (mBKS_timed (16)_) did not change test performances for the temporal agreement of both Off state and Dyskinetic state detection (Fig. 6a, b; Supplementry Fig. 7). For data on timing for the detection of participant-rated motor state, please refer to Supplementary Fig. 8.

## Discussion

The primary finding of this prospective observational multi-center cohort study in 63 participants with advanced PD is that the validity of wrist-wearable accelerometer-based digital Parkinson’s Motor Diary (adPMD) real-world monitoring of PDTs is moderate for Off time and Dyskinetic time when compared to simultaneous clinical rating (ICC = 0.43–0.51), while temporal agreement at a given time point on the half-hour time level is poor (median Cohen’s *κ* = 0.13–0.21). The accuracy between adPMD and observer were higher than those between adPMD and participant ratings when analyzing all motor states. Synchronizing adPMD data collection to the time of diary entries and using an index day to calibrate the adPMD threshold to patient diary entries did not increase the concordance between patient diary entries and adPMD monitoring. Calibration of adPMD data to clinician ratings improved their concordance of Dyskinetic state detection to the moderate level (Cohen’s *κ* = 0.41).

DHTs have been particularly developed because the features of PD that respond to dopaminergic stimulation fluctuate due to the shorter benefit from levodopa dosing in advanced disease^37^. Consequently, a single assessment fails to capture the presence, timing or severity of these fluctuations and so continuous assessment is required. While patient diaries were developed to address this, there have been misgivings about their accuracy^19,23,24^. For example, in the recent studies, subjects who collected diaries while under video or face-to-face clinical observation failed to report dyskinesia observed by the clinician in 34–64% of instances^19,23,24^. However, diaries, which classify motor function into three motor states^15,22,27,31,39–41^ have influenced the way these motor states are considered: The patient initially determines the level of bradykinesia that marks the transition from On to Off state. However, there is likely to be variation during the course of the day as to where that point of transition is perceived as well as variation between subjects. To compare the diary to an objective measurement, including UPDRS III, 7m-TUGT or DHTs, requires a point on that objective measurement to be used as the transition point. Although the observers and participants were intensively trained in diary rating, the application of the criteria for each motor state has inherent inconsistencies that introduce variability into an individual rater’s scores and between raters. Furthermore, there is no objective threshold to aid the rater in determining when a switch between motor state categories has occured. Clinical measures of bradykinesia/dyskinesia, such as UPDRS^16^ or mAIMS^17^, which closely correlate with PKG^®^ scores^29,38–40^, are more quantitative and would permit a threshold. However, we chose not to use them because they would consume to much of each 30-min interval and may potentially interfere with PKG^®^ recordings.

We used the PKG^®^ device^29–34^ in an alternative application format by converting its continuous data into one of the three motor categories of the PD home diary on a half-hour basis^15,22,31,41^ (resulting in adPMD ratings) and analyze its validity using half-hourly clinical ratings as the main validation criterion. We not only analyzed the PDTs as most previous studies^27,39,40^, but we were also interested in the temporal agreement of DHT data and clinical ratings at a given time point and during motor Off episodes to detect unpredictable day-to-day differences^49^. Our study settings were as uncontrolled as possible when testing the motor function each half-hour during the waking day, but the frequent ratings and manual diary entries might have nevertheless interfered with motor function. We demonstrated moderate validity for Off time and Dyskinetic time, but only limited correlation/validity for the other measures of motor fluctuations. In particular, temporal agreements between adPMD assessments and simultaneous observer ratings are limited, which can lead to unpredictable Off or Dyskinetic episodes being overlooked in clinical routine.

While our main outside validation criterion assessed by clinical raters overcome the numerous problems with patient diaries including individual selecting their own (possibly varying) threshold, interference with non-motor aspects, diary fatigue and others (see detailed discussion in Ossig and colleagues^31^), clinical examinations are also associated with limitations and inter-rater agreement data are lacking. Even though the clinical raters took global bradykinesia, tremor, dyskinesia and gait function into account, they were not required to score according to specific thresholds or cut-off scores such as to score Off state with reference to a specific UPDRS III score (noting though there was insufficient time for a formal UPDRS III assessment). Similarly, there was no decision about what number of seconds performing a 7m-TUGT constituted Off state. However, we validated observer diary ratings against the results of a simultaneously adjudicated 7m-TUGT^23^: Observer clinical diaries showed better separation of 7m-TUGT results between Off and On states and a lower variability during On without dyskinesia as compared to PD home diary ratings arguing for a higher consistency and reliability of observer ratings. Consequently, we attempted to calibrate the scoring of clinical observers and diaries against the PKG^®^ standard threshold. Our initial analyses thus compared uncalibrated adPMD data with observer and participant diary data and thereby confirmed PKG^®^ standard threshold or target values^29^, but with rather weak discriminatory power. This poor overall temporal agreement between adPMD data and observer ratings prompted us to test various strategies to optimize the cut-offs: We repeated all analyses with adPMD data calibrated to the patient’s individual thresholds^31^ without relevant changes in validity measures. We next tested the effects of individualizing cut-offs according to the clinician ratings as another approach for calibrating adPMD data by using ROC analyses. The first important observation was that only in 36% of participants the detection of Off states and in 56% the detection of Dyskinetic states are successful. However, the use of individual cut-off values in the whole cohort leads to an increase of the validity of adPMD data, which is even further increased by selecting participants with significant ROC results. The wide ranges of cut-off values in combination with the wide ranges of corresponding Youden indices for both Off state and Dyskinetic state detection further demonstrate the importance of an individualized transfer of DHT data into motor states. Unfortunately, we did not find clinical predictors to identify the individuals with significant motor state detection by the DHT. Since we did not predefine the analytic strategy using individualized cut-offs in our study protocol, the data on thresholding of PKG^®^ data transfer into adPMD motor states needs to be confirmed in a future prospective trial.

To assess whether DHT provides an accurate assessment of the motor states at a particular point in time, the digital monitoring must temporally coincide with the clinical assessment. This forces constraints on digital monitoring that do not easily fit with the design of the PKG^®^. As the PKG^®^ produces a score every 2 min, notionally that 2-min score could be used to compare with an assessment done contemporaneously. Moreover, best results are obtained from that point in the day being represented by a smoothed moving average (median) over at least 30 min (15 × 2-min epochs) during usual activity which is then averaged again over the same period from 6 days. An important reason for longer periods of monitoring and the use of medians is that it reduces the likelihood of atypical physical activity or inactivity biasing the score. When only 30-minutes time periods are used, the benefit of this averaging is lost using the method adopted in the present study.

The short time period of 30 min applied as the clinical reference period requires exact timing of simultaneous DAT recordings and clinical ratings. We thus examined—in addition to standard 30-min intervals used in the total cohort—two different exactly timed periods for assessing adPMD data excluding the clinical testing time period, each differing in their relation to the time of the clinical rating: One 28 min period exactly around clinical testing, and the second one was a 16-min period directly prior to clinical ratings, which was thought to more likely depict the motor state present when the observer made their clinical ratings. In keeping with the design of the PKG^®^ algorithms, the median of the 2-min epochs were used to produce mBKS and mDKS scores. The correlation between the adPMD data from the two periods and standard recordings for both Off state and Dyskinetic state was similar.

Our validation data are in good agreement with previous reports using passive uncontrolled assessment of motor function by DHTs in comparison to clinical rating scales, such as the MDS-UPDRS (present data and^27,50^). However, these scales do not necessarily reflect motor states at a given time point, but more closely the PDTs or even an average over several days. The higher correlations or validity measures in many previous studies using various wearables including STAT-ON, PKG^®^ and the Verily smartwatch with partly excellent validity indices^29,35,39,40,51^, are most likely related to notable differences in the study designs: They used more controlled settings or even a virtual exam for their assessments, in some cases combined with averaging the DHT signals over several hours or even days to reduce the effects of interference of the DHT signal with voluntary motor activity/inactivity^29,35,39,40^. As a limitation of our study, we did not record (voluntary) mobility of the participants during PKG^®^ recordings to enable the investigation of this interference. Indeed, most current DHT systems use as a key idea a statistical approach of measuring multiple times. In this view, the central tendency becomes the likely state of bradykinesia or dyskinesia of the sample, and the variation determines the confidence of that measure. The systems thus measure under conditions of lower variation (controlled conditions) or increase the sample size by recording for several days and between 09:00 and 18:00 (to reduce times of inactivity). This general approach leads not only to a close correlation of mBKS or other DHT measures of bradykinesia and conventional clinical rating scales such as the UPDRS part III motor score but also to a clear response to levodopa medication^29,38–40^. Unfortunately, we did not assess the time points of medication intake to assist the interpretation of our data. The available data also imply that even though the PKG^®^ is placed on only one wrist it is able to estimate motor dysfunction in PD when used in its standard format^29–34^. However, it cannot be entirely excluded that a single wrist-worn device might not be able to capture full anatomical distribution of symptoms emerging during transitions between motor states and thus needed to detect the various motor states by the alternative adPMD approach. Interference of the adPMD dyskinesia rating with tremor is unlikely in our cohort, because less than 2% of participants showed mild or moderate tremor of the upper extremities. Moreover, DHTs including the PKG^®^ reliably detect tremor and differentiate tremor from dyskinesia^50,52,53^. Together, our data suggest that the combination of frequent virtual examination (active assessment under controlled conditions) and passive monitoring of motor function might potentially help to increase validity of DHTs in detecting motor fluctuations in remote real-world scenarios.

In conclusion, the adPMD using the PKG^®^ device captures daily times in Off and Dyskinetic state assessed using the PD home diary in fluctuating PD with moderate validities as determined by ICC analyses. Limited validity of adPMD data with respect to temporal agreement of motor state ratings at a given time point limits its use for detecting unpredictable Off episodes. The loss of benefit by not smoothing/averaging the DHT signal seems an important factor for this limitation. Our data suggest that the combination of frequent virtual examination (active assessment under controlled conditions) and passive monitoring of motor function might potentially help to increase validity of DHTs in detecting motor fluctuations in remote real-world scenarios. Future studies in larger patient cohorts are warranted to confirm our data on thresholding for PKG^®^ data transfer into motor state diary data and to evidence the adPMD using the PKG^®^ device as a suitable trial endpoint.

## Participants and methods

### Study protocol approvals and participant consents

This study reports the prospective, observational VALIDATE-PD cohort study^23,24^ on alternative application of the wrist-wearable accelerometer Parkinon KinetiGraph^®^ (PKG^®^; Global Kinetics, Melbourne, Australia) for objective motor diary assessment (wrist-wearable accelerometer-based digital Parkinson’s Motor Diary, adPMD). The study was conducted at three hospital centers in Germany and Sweden (University Medicine Rostock, Germany; Movement Disorder Clinic Beelitz-Heilstätten, Germany; Neurology Research Unit, Skåne University Hospital, Lund, Sweden) as described previously^23,24^. The study was approved by the institutional review boards of all participating centers (ethic committee registry numbers A 2017-0115 for Rostock, AS 84(bB)/2018 for Beelitz-Heilstätten and the Regional Ethics Review Board, Lund, Sweden (2017/936). All participants gave written informed consent to participate in the study and were advised both orally and in writing of the nature of the study.

### Participants

Participants were eligible for the study if they were over 30 years old, had been diagnosed with PD according to the United Kingdom PD Society Brain Bank criteria, suffered from motor fluctuations observed by the treating physician and/or documented on part 4 of the Movement Disorder Society-revised Unified Parkinson’s Disease Rating Scale (MDS-UPDRS) and were able to provide written informed consent.

Exclusion criteria comprised the existence of clinical signs for secondary or atypical parkinsonian syndromes, inability to complete questionnaires and/or patient diaries, lack of cooperation during the study procedures, presence of dementia (defined as scores on the Montreal Cognitive assessment [MoCA] < 21)^54^ and/or relevant psychotic symptoms, ongoing treatment with advanced/invasive therapies (deep brain stimulation, subcutaneous apomorphine and levodopa-carbidopa intestinal gel).

We screened 96 participants for eligibility of whom 91 were successfully included into the present study. Four participants were excluded because of MoCA scores below 21 points at screening (*n* = 2) or were not able to sufficiently adhere to diary assessments (*n* = 2). One participant declined further participation due to undisclosed reasons after the screening visit. Of these 91 participants, 63 participants (69%) had 2 days of calibrated adPMD datasets and were included in the final study analyses (for 5 participants, no recordings at day 0 were availble, for 14 participants only 1 day of PKG^®^ recordings were available and for 9 participants less then 30 half-hours (<71%) of observer readings or PKG^®^ recording periods were available). According to MDS-UPDRS part IV, participants reported a median of 18% (IQR: 7–28%) time in Off state, 65% (50–82%) in On state and 13% (6–29%) in Dyskinetic state. Although tremor was not an exclusion criterion, only 1.6% of participants showed mild or moderate resting/postural/action tremor of the upper extremities (MDS-UPDRS tremor items 3.15–3.17).

### Clinical assessments

We assessed basic demographic and clinical data including PD medication, clinical phenotype, type of motor complication, Hoehn-Yahr score^55^, MDS-UPDRS^56^, cognitive screening with MoCA^54^ and Beck’s Depression Inventory version 2 (BDI-II)^57^. After inclusion into the study, all participants received detailed instructions on the PD home diary^15,22,41^. Participants of the German centers additionally watched a training video explaining all functional states with particular focus on the difference between tremor and dyskinesia^41^. Participants were then asked to indicate their predominant status during half-hour time periods for 3 days using the categories Asleep, Off (herein called Off state), On without dyskinesia (On state), On with non-troublesome dyskinesia, and On with troublesome dyskinesia using the PD home diary)^15^. While diary data for both participants and the observer was initially collected using these categories^15^, we eventually combined the categories “On with non-troublesome dyskinesia” and “On with troublesome dyskinesia” into the category “On with dyskinesia” for analysis (Dyskinetic state), since the distinction between non-troublesome and troublesome dyskinesia could only be made by participants, but neither by observers nor the adPMD. In the present study, we only used PD home diary data from the initial day (day 0) for adPMD calibration (see below). On the following 2 days (days 1 and 2), participants were observed by experienced PD raters (A.B., J.T. and acknowledged personnel), who had been trained to identify motor complications in advanced PD patients in the participating hospitals and acquired MDS certification as qualified UPDRS raters prior to study start. The observers/clinical raters independently evaluated motor states half-hourly throughout daytime (8 a.m. through max. 6 pm) based on clinical observations taking global bradykinesia, tremor, dyskinesia and gait function into account. In the German cohort, 7-meter version of the Timed-Up-and-Go test (7m-TUGT) times^58^ were recorded but not considered for observer ratings. The exact time points of the clinical rating (synchronized time with the PKG^®^ device) was documented in the German cohort. The 7m-TUGT was canceled at 1 min for patient safety and to not interfere too much with the real-world monitoring environment (total clinical rating and diary assessment time not exceeding 1:30 to 2:00 min). The observer diary ratings were used as the outside criterion for all validity analyses and PD home diary data were used for ancillary analyses (see Table 2 for glossary of clinical ratings).

### adPMD assessments

We used the PKG^®^ system as a wrist-worn accelerometer-based device, which was worn at the wrist from day 0 until day 2 on the side more severely affected by PD. The primary outcome measures of the PKG^®^ are the continuous quantitative bradykinesia scores (BKS) and dyskinesia scores (DKS) for 2-minute time periods arising from the analyzing technique of the spectral power of the low frequencies of accelerometer data as developed by Griffith et al.^29^. For the alternative application of the adPMD, we calculated the 30 min median BKS with times of inactivity/non-wrist wear excluded (mBKS) and the 30 min median DKS with times of activity excluded (mDKS; see Table 2 for definitions of PKG^®^ and adPMD measures). The algorithms used to transfer these quantitative measures into motor states were already introduced by ref. ^31^. These algorithms convert each half-hour of continuous quantitative mBKS/mDKS into one of the five categories (asleep, motor Off state, On state or On state with dyskinesia [Dyskinetic state], not wearing PKG^®^ device) as used in the observer diary and the PD home diary^23,31^. In brief, the 75^th^ percentiles of BKS and DKS of normal subjects were used from data of the study by Griffith and colleagues^29^ and the probability that more BKS or DKS was greater than the 75^th^ percentile of controls in each half-hour period was estimated using the χ^2^ test. There are fifteen 2 min epochs in a half-hour and in normal subjects, one in four will be over the 75^th^ percentile of controls. Thus in patients, if 15 or more BKS or DKS are greater than 75^th^ percentile of controls in the hour then the χ^2^ test provides a *P* value < 0.05. These data are herein referred to as uncalibrated adPMD motor state data. As well as correlating adPMD data with the consistent threshold, we additionally calibrated adPMD data according to Ossig and colleagues by modeling the thresholds used by the individual participant^31^. To achieve this, uncalibrated adPMD data were tuned to model day 0 of diary entries by each patient (referred herein as adPMD [calibrated] data) for establishing individual thresholds for Off and Dyskinetic state. Calibration from that first day (day 0) was then applied to the subsequent 2 days. adPMD analyzers were blinded to clinical data and observer/patient diary entries (H.K., N.S., M.H.).

For further analyses, we used exactly timed PKG^®^ recordings from the German cohort for diary classification using the adPMD method with time frames from the 28 min around the clinical ratings (seven 2-min epochs before and seven epochs after clinical rating, separated by one 2-min epoch during which the clinical testing took place; mBKS/DKS_timed (28)_) or the 16 min directly prior to clinical ratings (eight 2-min epochs before rating; mBKS_timed (16)_).

### Adherence to adPMD and clinical diary assessments

All VALIDATE-PD study participants who had 2 days of calibrated adPMD datasets with at least 30 half-hour time periods (>70% of all half-hour periods between 8:00 and 18:00) with simultaneous adPMD and observer diary data were included in final study analyses leading to analyzable datasets from a total of 63 participants (German cohort: *n* = 40).

In total, we detected 2435 half-hour periods during the waking day with observer diary entries (92.0% of all half-hour periods between 8 a.m. and 6 p.m.). 32 time periods (1.2% of all time periods) rated as Asleep by the observers were excluded from further analyses. From participant diaries, we analyzed 2443 (92.3%) half-hour periods with 40 (1.6%) half-hour periods being excluded due to the rating Asleep. In the German cohort, we generated 1613 half-hour periods (95.4% of all periods) of timed clinical and adPMD assessments and further performed 1566 half-hourly 7m-TUGTs (92.6% of all half-hour periods) used as a clinical measure of bradykinesia^59^. We further detected 2444 (92.4%) half-hour periods with adPMD scores, and 2384 (90.1% of all periods or 97.5% of all PKG^®^ scorings) half-hour adPMD scores were classifiable into motor states with 144 periods (6.0%) classified as Asleep, 974 (40.9%) as motor Off state, 780 (32.7%) as On state and 486 (20.4%) as Dyskinetic state. Calibration was possible in 1944 half-hour periods (73.5% of all periods or 79.5% of all adPMD scorings) with 115 periods (5.9%) classified as Asleep, 566 (29.1%) as motor Off state, 600 (30.9%) as On state, and 663 (34.1%) as Dyskinetic state.

These numbers translated into 2339 half-hour periods (88.4%) with complete simultaneous ratings of motor states in observer diaries and uncalibrated adPMD data, and 1877 (70.9%) half-hour period with simultanous observer-ratings and calibrated adPMD data. For participant diaries, we analyzed 2338 half-hour periods (88.4%) with complete simultaneous ratings of motor states, and 1913 (72.3%) half-hour period with simultanous participant-ratings and calibrated adPMD data. Simultaneous timed assessments and 7m-TUGT were available for 1361 (81.0%) half-hour periods.

For analyses on the participant level, we used PDTs of the three different motor states (time in motor state per total time from 8 a.m. to 6 p.m.) of 2 consecutive days from 63 participants with sufficient datasets from two days with a median of 42 (IQR: 34–42) half-hour datasets or 100% (81–100%) of total datasets for clinical ratings and 40 (IQR: 34–42) half-hour datasets or 95% (81–100%) of total datasets for adPMD recordings. Separate analyses of single day values revealed similar results (data not shown).

### Data analyses and statistics

Statistical analyses were performed using IBM SPSS Statistics software version 27 and Excel (IBM Corporation, New York, USA). Values are provided as numbers (percentages) or median (interquartile range, IQR), as appropriate. Boxplots are shown with a central mark at the median, bottom, and top edges of the boxes at 25th and 75th percentiles, respectively, whiskers out to the most extreme points within 1.5 times the interquartile range, and outliers scoring more than 1.5 × IQR but at most 3 × IQR outside the quartiles. Pairwise exclusion was used for missing values. As most of the data were not normally distributed, we chose non-parametric tests for statistical comparisons such as Kruskal-Wallis tests with Dunn-Bonferroni post-hoc tests, corrected for multiple comparisons, or Friedman tests with post-hoc Wilcoxon signed-rank tests with Bonferroni correction for multiple comparisons. *P* values < 0.05 (two-tailed) were considered to be statistically significant.

### Test performance of adPMD data for the detection of diary motor states

Statistical analysis of the three motor states (motor Off, On or On with dyskinesia) from clinical observer or PD home the diary data was then confined to all half-hour periods between 8 a.m. and 6 p.m., for which simultaneous ratings from adPMD and observers had been recorded. Assessments on the half-hour time level used the following preformance measures from the 2 × 2 contingency tables: Sensitivity (or recall) expressed in percent, specificity in percent, accuracy in percent^60^, balanced accuracy as the average of sensitivity and specificity in percent^61^, precision in percent, Cohen’s *κ*^62^, Matthews correlation coefficient (MCC or *Φ* coefficient)^60,62,63^ and F1-score as the harmonic mean of sensitivity and precision^60^. Imbalance of outcome classes was estimated as the imbalance ratio (IR) defined as the number of majority class samples (negative ratings) divided by the number of minority class samples (positive ratings) and values of 5 or higher were considered as relevant (moderate to severe) imbalance^64^. Pearson’s correlation test and intraclass correlation coefficient (ICC) estimation were used for correlations of daily times spent in the various motor states on the participant level. Pearson’s correlation coefficient │*r*│ < 0.3 was considered a weak, │*r*│ = 0.3–0.59 a moderate and │*r*│ ≥ 0.6 a strong correlation. ICC estimates and their 95% confidence intervals (95%CIs) were calculated based on single-rating, absolute-agreement, 2-way mixed-effects models with two rating instruments across all participants. According to the guideline by Cichetti^65^, we interpret *κ* values or ICC < 0.40 as poor, *κ*/ICC = 0.40–0.59 as moderate, *κ*/ICC = 0.60–0.74 as good and *κ*/ICC = 0.75–1.00 as excellent validity.

### Data balancing

Although diary outcome data were not or only mildly imbalanced with IR values between 1.56 and 2.94 for clinical observer diary data and 1.01 and 3.45 for PD home diary data^64^, we addressed the challenge of class imbalance by balancing the data using random undersampling the majority class, random oversampling the minority class and combined under-/oversampling of clinical observer and PD home diary data^64,66^. Analyses of balanced datasets revealed no relevant influence of data imbalance in the original datasets with stable values for major performance measures such as sensitivity, specificity, balanced accuracy, Cohen’s κ and MCC (for details, refer to Supplementary Results).

### Association of diary data with demographic and clinical factors

Pearson’s correlation test and multiple linear regression modeling was used to test the association of demographic and clinical candidate factors with adPMD quantitative scores and motor state results as well as with the agreement rates and Cohen’ κ values for adPMD motor state results with observer-rated diary entries. We included the variables age, sex, symptom duration, MDS-UPDRS part III motor score as a measure of disease severity, motor fluctuation duration, MDS-UPDRS part IV as a quantitative measure of motor fluctuations, BDI and MoCA as independent covariates into the models using a step-wise selection with *P* < 0.05 for adding and *P* < 0.10 for removing variables. Multicollinearity of candidate variables were excluded by Pearson correlation test (|*r*| < 0.5). Results were confirmed by hierarchical multiple linear regression models.

### Temporal connection between Off episodes

For analyzing the temporal connection between Off episodes as rated by the clinical observers, Off episodes (defined herein as an motor Off state of at least 30 min duration following a motor On period of at least 90 min) from all participants were synchronized by summation of all Off state periods as rated by observers using the first 30 min of the motor Off period as the trigger event (start of Off episode). The Off state ratings from the adPMD were then cross-classified by putting them into 2 × 2 contingency tables for each 30-min motor Off state interval. All diary sets were included for analysis. Individual time periods were excluded from analysis if there was no response or more than one response on either diary.

### Association of quantitative PKG^®^ scores with diary data

For analyses of quantitative PKG^®^ scores^29^, we initially compared mBKS and mDKS with the corresponding diary data and 7m-TUGT results (used as a quantitative measure of bradykinesia^58^) on the half-hour time period level. Consistently, we detected significantly higher mBKS and higher 7m-TUGT times during Off state periods as compared to On state and Dyskinetic state periods as assessed by the clinical observers, while mDKS were lower during Off state periods compared to On state and Dyskinetic state periods as assessed by clinical observer diaries (*P* < 0.001, Kruskal-Wallis tests). Further analyses of quantitative PKG^®^ scores are reported in Supplementary Results.

### Receiver operating curve (ROC) and precision-recall curve (PRC) analyses of PKG^®^ scores

ROC analyses were used to display the correlation between specificity and sensitivity, and PRC plotting sensitivity/recall versus precision to display the fraction of true positives among positive detections of the quantitative PKG^®^ scores for detection of motor states (mBKS for Off states and mDKS for Dyskinetic states). The area under the curve (AUC) with 95% confidence intervals (95%CI) was used to compare the various models (AUCs with 95%CIs of PRC plots were calculated using the trapezoid rule with the assumption of normal data distribution with the SPSS data output). In ROC analyses, a relevant discrimination of motor states by PKG^®^ scores was defined as an ROC-AUC significantly larger than 0.5. The maximal Youden index (J = Sensitivity + Specificity – 1) was used to estimate optimal cut-off values. PRC plots with their AUCs using as an overall performance measure express the susceptibility of the classifiers to imbalance of the data and was thus applied as an accurate comparison of the classifier performance between datasets with difference imbalance ratios^67^. The results of PRC plot analyses are displayed in Supplementary Results.

### Reporting summary

Further information on research design is available in the Nature Research Reporting Summary linked to this article.

### Supplementary information

Supplementary Information

Reporting Summary

## Data Availability

The main data supporting our results in this study are almost all available in the manuscript and Supplementary Information. We are sorry that the data from the hospitals cannot be made publicly available because of hospital regulation restrictions and privacy concerns to protect our patients. Anonymized data might be accessible for research purposes from the corresponding authors upon reasonable request.
