# Supplementary material for: Application of single wrist-wearable accelerometry for objective motor diary assessment in fluctuating Parkinson’s disease
Source: NPJ Digit Med. 2023 Oct 17;6:194. doi: 10.1038/s41746-023-00937-1 (PMC10582031; doi:10.1038/s41746-023-00937-1)
Supplement: Supplementary file 1 — Supplementary Information [file 41746_2023_937_MOESM1_ESM.pdf]

Supplementary Materials

Application of wrist-wearable accelerometry for objective motor diary assessment in fluctuating Parkinson's disease

Matthias Löhle, Jonathan Timpka, Alexander Bremer, Hamid Khodakarami, Florin Gandor, Malcome Horne, Georg Ebersbach, Per Odin and Alexander Storch

Supplementary Text:

- **Supplementary Results.** Agreement of adPMD motor diary classification with participant diary ratings during Off episodes.
- **Supplementary Results.** Precision-recall curve (PRC) plot analyses.
- **Supplementary Results.** Potential factors predicting individual thresholding for adPMD diary ratings.
- **Supplementary Results.** Data imbalance and adPMD test validity measures.
- **Supplementary Results.** Association of quantitative PKG® scores with observer diary motor states data and 7m-TUGT results.
- **Supplementary Results.** Association of quantitative PKG® scores with participant diary motor states data and 7m-TUGT results.

Supplementary Tables:

- **Supplementary Table 1.** Validity parameters of the adPMD for the detection of clinical observer ratings according to the various cut-offs as determined by ROC plot analyses for transferring PKG® scores into adPMD states.
- **Supplementary Table 2.** Validity parameters of the adPMD for the detection of participant PD Home diary ratings according to the various cut-offs as determined by ROC plot analyses for transferring PKG® scores into adPMD states.
- **Supplementary Table 3.** Correlations and multivariate regression analyses of candidate factors associated with PKG daily motor state times.
- **Supplementary Table 4.** Test Validity parameters of the adPMD for the detection of clinical observer Off ratings in balanced datasets.
- **Supplementary Table 5.** Validity parameters of the adPMD for the detection of clinical observer On ratings in balanced datasets.

- **Supplementary Table 6.** Validity parameters of the adPMD for the detection of clinical observer Dyskinetic ratings in balanced datasets.

Supplementary Figures:

- **Supplementary Figure 1.** Preferred choices in observer- and participant-documented diaries in the respective calibrated adPMD motor states.
- **Supplementary Figure 2.** Temporal agreement of observer- and participant-documented data and adPMD data on the participant level.
- **Supplementary Figure 3.** Temporal agreement between PKG® scores, 7m-TUGT results and observer and patient-documented motor states over the time course of Off episodes.
- **Supplementary Figure 4.** Temporal agreement between observer and participant diaries and adPMD ratings over the time course of Off episodes.
- **Supplementary Figure 5.** Precision-recall curve (PRC) plot analyses of PKG® quantitative scores for the detection of participant-documented Off and Dyskinetic state.
- **Supplementary Figure 6.** Test performance of PKG® scores for the detection of observer-documented Off and Dyskinetic state using individualized cut-off values.
- **Supplementary Figure 7.** Test performance of timed PKG® scores for the detection of observer-documented Off and Dyskinetic state.
- **Supplementary Figure 8.** Test performance of PKG® scores and 7m-TUGT results for the detection of participant-documented Off and Dyskinetic state.
- **Supplementary Figure 9**. Correlation of observer-rated diaries and 7 meter timed-up-and-go test data with PKG® bradykinesia scores (mBKS) on the half-hour time period level.
- **Supplementary Figure 10**. Correlation of observer-rated diaries and 7 meter timed-up-and-go test data with clustered PKG® bradykinesia scores (mBKS) on the half-hour time period level.
- **Supplementary Figure 11.** Correlation of observer-rated diaries and 7 meter timed-up-and-go test data with PKG® dyskinesia scores (mDKS) on the half-hour time period level.
- **Supplementary Figure 12.** Correlation of participant diaries and 7 meter timed-up-and-go test data with PKG® quantitative scores on the half-hour time period level.

SUPPLEMENTARY RESULTS

Agreement of adPMD classification with participant diary ratings during Off episodes

We estimated the sensitivities of the wearable accelerometer-based digital Parkinson's Motor Diary (adPMD) ratings for the detection of participant PD Home diary ratings with respect to time course of Off episodes, we used the the approach of synchronizing Off episodes (***Supplementary Figs. 4c-f***). Taking the participant-rated diary data as the outside criterion, sensitivities of the adPMD ratings during the first 2.5 hours of the Off episodes ranged from 51% to 64% (balanced accuracy range: 63-74%) with no major differences over the time course of Off episodes. Calibration of adPMD data to participant's individual thresholds did not lead to relevant differences of agreement rates (***Supplementary Figs. 4e,f***).

Precision-recall curve (PRC) plot analyses

We report the precision-recall curve (PRC) plot (plotting sensitivity/recall versus precision) analyses in addition to the ROC analyses, because PRC plots are believed to be more informative/reliable in imbalanced datasets[1]. Indeed, ROC-curves might be too optimistic in imbalanced datasets[1]. To compare PRC plot results, we calculated the PRC-AUC including their 95%CI using the trapezoid rule with the assumption of normal data distribution (***Supplementary Figure 5, Supplementary Table 1***). Indeed, the PRC plot results (PRC-AUC) better correlated with the test performance measures of the different adPMD applications as compared to ROC-AUC comparing standard cut-off, calibrated cut-off and individualized cut-off for all participants (see ***Supplementary Table 1*** for details).

Potential factors predicting individual thresholding for adPMD ratings

By searching for potential factors predicting significant discrimination of Off and Dyskinetic state by the respective PKG® scores, we did not detect any significant association of major demographic, clinical and diary parameters with and between the group of participants with significant discrimination and that without discrimination for both motor states ($P \geq 0.05$ from Mann-Whitney U-test or χ^2 test as appropriate; see ***Table 1*** for covariable list; data not shown). In agreement, there was no correlation of the individual AUC values from ROC analyses for detecting Off and Dyskinetic state by the respective PKG® scores with demographic or clinical variables ($P \geq 0.05$ from Mann-Withney U-test or Pearson correlation test as appropriate; see ***Table 1*** for coviarable list; data not shown). Moreoever, we did not observe any correlation of AUC values from ROC analyses for both descriminations with the magnitude of test outcomes (PDTs of the respective motor states) making a relevant proportional bias improbable (***Supplementary Figs. 6a,b***).

Data imbalance and adPMD test performance/validity measures

With class imbalance ratios (IRs) of the clinical outcome measures (IR: number of majority class samples / number of minority class samples) between 1.56 and 2.94 for the clinical observer diary data and 1.01 and 3.45 for the participant's PD home diary data, our datasets are not or only mildly imbalanced (although there is no general rule for the interpretation of class imbalance in the literature, an IR of 5 or higher is usually considered a moderate to severe imbalance)[2]. However, to address the influence of dataset imbalance on adPMD test performance analyses, we used two approaches: First, calculating additional test performance measures, which are largely independent of dataset imbalance, such as balanced accuracy[3] or Matthew correlation coefficient (MCC)[4-7]. Secondly, we balanced our datasets using three different approaches prior to calculating various test validity parameters.

Supplementary Table 1 shows major test performance measures of adPMD for the detection of clinical observer diary motor states and ***Supplementary Table 2*** for the detection of participant's PD Home diary motor states. As reported previously, within the positive range of Cohen's κ, Cohen's κ is strongly correlated to the MCC as a test performance parameter, which is rather insensitive to data imbalance[4-7]. Consistently, Cohen's κ values of the present study (all values were within the positive range) were nearly identical compared to their corresponding MCC values (***Supplementary Tables 1,2***).

We next balanced the test datasets with respect to the three clinical observer-documented motor states as well as to the PD home diary motor states using three different approaches (random undersampling, random oversampling and random combined under-/oversampling)[2,8]. Statistical analyses of balanced datasets in comparison to the original dataset revealed in general no relevant influence of data imbalance in the original datasets with stable values for major performance measures such as sensitivity (recall), specificity, balanced accuracy, Cohen's κ and MCC (see ***Supplementary Tables 4-6*** for exemplary results on adPMD test performance for the detection of clinical observer-documented motor states). As expected from the literature[6], accuracy, precision and the F1-score showed relevant dependence on data balancing.

Together, the major test performance/validity measures sensitivity, specificity, balanced accuracy and Cohen's κ were not relevantly influenced by the mild degree of dataset imbalance in the present study and thus meaningful for the interpretation of the test performance/validity of the adPMD.

Association of quantitative PKG® scores with observer diary data and 7m-TUGT results

We examined the quantitative PKG® scores[9] in comparison to the corresponding diary data and 7m-TUGT results on the half-hour time period level. We analyzed 2,339 half-hour periods (88.4% of all periods between 8 am and 6 pm) for the total cohort (n=63) and 1,361 (81.0%) for the German cohort (exactly timed PKG® recordings and 7m-TUGT assessments; n=40) during the waking day with complete simultaneous ratings of motor states in observer diaries and adPMD data (***Supplementary Fig. 9***). 615 (26.0% of all periods [27.2% in German cohort]) half-hour periods were classified by observers as Off state, 915 (38.7% [38.1% in German cohort]) as On state and 833 (32.3% [32.9% in German cohort]) as Dyskinetic state. We found significantly higher median PKG® bradykinesia scores (mBKS) and higher 7m-TUGT times during Off state periods as compared to On state and Dyskinetic state periods as assessed by the clinical observers (*P*<0.001, Kruskal-Wallis test).

Pearson correlation analyses of 7m-TUGT results with mBKS units revealed only a weak direct correlation (***Supplementary Fig. 9d***), and locally estimated scatterplot smoothing (LOESS) showed a ceiling effect for mBKS scores at 7m-TUGT results of >25 sec. Separate correlation analyses for Off state, On state and Dyskinetic state clusters revealed no major changes in correlation strengths. Correponding Bland-Altman plots showed a mild proportional bias with an increasing difference between both mBKS measures and 7m-TUGT results as the value of measurement increases (***Supplementary Fig. 9e***). Correlation analysis of 5 sec-7m-TUGT clusters of mBKS units with 7m-TUGT results revealed a higher Pearson correlation coeficient (r=0.82; *P*=0.004; ***Supplementary Fig. 10a***). Changing the time frames of mBKS recordings to exactly 28 min around the clinical ratings (seven 2-min-epoches before and 7 epoches after clinical rating) did in general not change the association of PKG® and clinical bradykinesia assessments (***Supplementary Figs. 9f,g, 10b***).

Median PKG® dyskinesia scores (mDKS) were lower during Off state periods compared to On state and Dyskinetic state periods as assessed by clinical observer diaries (*P*<0.001, Kruskal-Wallis test; ***Supplementary Fig. 11***).

Association of quantitative PKG® scores with participant diary motor states data and 7m-TUGT results

For comparing the PKG® data with PD home diary data, we examined the quantitative PKG® scores as the primary PKG® outcome measures[9] and 7m-TUGT results[10] in comparison to the responding PD home diary data as documented by study participants on the half-hour time period level. Grouping the PKG® results with respect to the corresponding entries in the PD

home diaries (2,338 entries or 88.4% of all half-hour periods), we found similar patterns for mBKS and 7m-TUGT results as detected for the observer ratings (***Supplementary Fig. 12***).

Supplementary References

1 Saito, T. & Rehmsmeier, M. The precision-recall plot is more informative than the ROC plot when evaluating binary classifiers on imbalanced datasets. *PLoS One* **10**, e0118432, doi:10.1371/journal.pone.0118432 (2015).

2 Kumar, V. *et al.* Addressing Binary Classification over Class Imbalanced Clinical Datasets Using Computationally Intelligent Techniques. *Healthcare (Basel)* **10**, doi:10.3390/healthcare10071293 (2022).

3 Wei, Q. & Dunbrack, R. L., Jr. The role of balanced training and testing data sets for binary classifiers in bioinformatics. *PLoS One* **8**, e67863, doi:10.1371/journal.pone.0067863 (2013).

4 Chicco, D., Totsch, N. & Jurman, G. The Matthews correlation coefficient (MCC) is more reliable than balanced accuracy, bookmaker informedness, and markedness in two-class confusion matrix evaluation. *BioData Min* **14**, 13, doi:10.1186/s13040-021-00244-z (2021).

5 Chicco, D., Warrens, M. J. & Jurman, G. The Matthews Correlation Coefficient (MCC) is More Informative Than Cohen's Kappa and Brier Score in Binary Classification Assessment. *IEEE Access* **9**, 78368-78381 (2021).

6 Chicco, D. & Jurman, G. The advantages of the Matthews correlation coefficient (MCC) over F1 score and accuracy in binary classification evaluation. *BMC Genomics* **21**, 6, doi:10.1186/s12864-019-6413-7 (2020).

7 Boughorbel, S., Jarray, F. & El-Anbari, M. Optimal classifier for imbalanced data using Matthews Correlation Coefficient metric. *PLoS One* **12**, e0177678, doi:10.1371/journal.pone.0177678 (2017).

8 Lemaitre, A., Nogueira, F. & Aridas, C. K. Jmbalanced-learn: A Python Toolbox to Tackle the Curse of Imbalanced Datasets in Machine Learning. *J. Machine Learn. Res.earch* **18** (2017).

9 Griffiths, R. I. *et al.* Automated assessment of bradykinesia and dyskinesia in Parkinson's disease. *J Parkinsons Dis* **2**, 47-55, doi:10.3233/JPD-2012-11071 (2012).

10 Podsiadlo, D. & Richardson, S. The timed "Up & Go": a test of basic functional mobility for frail elderly persons. *J Am Geriatr Soc* **39**, 142-148, doi:10.1111/j.1532-5415.1991.tb01616.x (1991).

Supplementary Table 1. Validity parameters of the adPMD for the detection of clinical observer ratings according to the various cut-offs as determined by ROC plot analyses for transferring PKG® scores into adPMD states.

	Precision-recall curve-AUC (95%CI)	adPMD test performance measures from 2×2 contingency tables							
		Accuracy (%)	Balanced accuracy (%)	Sensitivity, recall (%)	Specificity (%)	Precision (%)	Cohen's κ	MCC	F1-score
Discrimination of Off versus Non-Off state from simultaneous clinical ratings									
mBKS (standard cut-off)	0.307 (0.281-0.333)	66%	65%	64%	67%	40%	0.26	0.27	0.49
mBKS (calibrated cut-off)	0.510 (0.480-0.539)	72%	66%	53%	79%	47%	0.30	0.31	0.50
mBKS (individualized cut-offs, all participants)[a]	0.542 (0.513-0.571)	71%	68%	62%	74%	44%	0.31	0.32	0.51
mBKS (individualized cut-offs, participants with sig. ROC only)[a,b]	0.349 (0.313-0.385)	77%	75%	70%	80%	62%	0.48	0.48	0.66
PD home diary (Off state)	-	26%	77%	63%	91%	71%	0.56	0.56	0.67
Discrimination of On state versus Non-On state from simultaneous clinical ratings									
Standard cut-off	-	56%	52%	38%	69%	42%	0.05	0.05	0.39
Calibrated cut-off	-	61%	56%	39%	73%	46%	0.13	0.13	0.43
PD home diary (On state)	-	65%	66%	70%	62%	54%	0.31	0.32	0.61
Discrimination of Dyskinetic state versus Non-Dyskinetic state from simultaneous clinical ratings									
mDKS (standard cut-off)	0.432 (0.407-0.456)	72%	65%	40%	90%	68%	0.34	0.35	0.50
mDKS (calibrated cut-off)	0.463 (0.436-0.490)	70%	67%	56%	78%	60%	0.35	0.34	0.57
mDKS (individualized cut-offs, all participants)[a]	0.542 (0.516-0.568)	71%	72%	75%	69%	59%	0.41	0.42	0.66
mDKS (individualized cut-offs, participants with sig. ROC only)[a,b]	0.562 (0.528-0.596)	79%	79%	74%	83%	78%	0.57	0.57	0.76
PD home diary (Dyskinetic state)	-	73%	68%	49%	86%	66%	0.38	0.39	0.57

Sensitivities, specificities, balanced accuracies and Cohen's κ values are in part from Table 3 to allow for easy comparison of test performance/validity measures.

[a]Individual cut-offs were estimated by ROC analysis for each participant for the discrimination between observer Off state and Non-Off state by mBKS and between observer-documented Dyskinetic and Non-Dyskinetic state by mDKS.

[b]Only participants with significant discrimination in ROC analyses (AUC significantly larger than 0.5) between observer Off state and Non-Off state by mBKS (n=20) and between observer-documented Dyskinetic and Non-Dyskinetic state by mDKS (n=29) were used for these analyses (and thus not included in comparative statistics).

Supplementary Table 2. Validity parameters of the adPMD for the detection of participant PD Home diary ratings according to the various cut-offs as determined by ROC plot analyses for transferring PKG® scores into adPMD states.

	Accuracy (%)	Balanced accuracy (%)	Sensitivity, recall (%)	Specificity (%)	Precision (%)	Cohen's κ	MCC	F1-score
Discrimination of Off versus Non-Off state from simultaneous PD home diary ratings								
mBKS (standard cut-off)	64%	63%	57%	65%	34%	0.21	0.22	0.44
mBKS (calibrated cut-off)	72%	64%	51%	78%	42%	0.27	0.27	0.46
Discrimination of On state versus Non-On state from simultaneous PD home diary ratings								
Standard cut-off	51%	51%	34%	68%	52%	0.02	0.02	0.41
Calibrated cut-off	58%	57%	37%	77%	58%	0.14	0.15	0.45
Discrimination of Dyskinetic state versus Non-dyskinetic state from simultaneous PD home diary ratings								
mDKS (standard cut-off)	71%	59%	34%	84%	44%	0.20	0.20	0.39
mDKS (calibrated cut-off)	69%	65%	57%	73%	44%	0.27	0.28	0.49

Supplementary Table 3. Correlations and multivariate regression analyses of candidate factors associated with daily motor state times from accelerometer-based digital Parkinson's Motor Diary (adPMD).

	Association with single candidate clinical factors		Multivariate regression model analysis[$]	
	Pearson correlation (r, P<0.05)[§]	Sex (P<0.05)[#]	Regression with observer data	Significant determinants (P<0.05)
adPMD daily motor state times with observer-rated diaries as variable of interest in regression analyses				
adPMD (uncalibrated)				
Off state	-	-	R^2=0.431, F=2.861, P=0.013	Observer-rated Off: β=0.408, P=0.015
On state	-	-	R^2=0.332, F=1.878, P=0.090	-
Dyskinetic state	MDS-UPDRS III: -0.265 (P=0.013)	P=0.002	R^2=0.616, F=6.067, P<0.0001	Observer-rated dyskinetic state: β=0.678, P<0.0001
adPMD (calibrated)				
Off state	MDS-UPDRS IV: 0.370 (P=0.002)	-	R^2=0.470, F=2.853, P=0.015	Observer-rated Off: β=0.602, P=0.001
On state	-	P=0.021	R^2=0.298, F=1.370, P=0.247	-
Dyskinetic state	MDS-UPDRS IV: -0.318 (P=0.009)	-	R^2=0.363, F=1.835, P=0.104	-
adPMD daily motor state times with patient-rated diaries as variable of interest in regression analyses				
adPMD (uncalibrated)				
Off state	-	-	R^2=0.420, F=2.654, P=0.020	MDS-UPDRS III: β=0.394, P=0.009
On state	-	-	R^2=0.264, F=1.315, P=0.267	-
Dyskinetic state	MDS-UPDRS III: -0.265 (P=0.013)	P=0.002	R^2=0.442, F=2.900, P=0.012	Patient-rated dyskinetic state: β=0.488, P=0.002
adP,MD (calibrated)				
Off state	MDS-UPDRS IV: 0.370 (P=0.002)	-	R^2=0.418, F=2.315, P=0.042	Patient-rated Off: β=0.560, P=0.005
On state	-	P=0.021	R^2=0.216, F=0.887, P=0.548	-
Dyskinetic state	MDS-UPDRS IV: -0.318 (P=0.009)	-	R^2=0.289, F=1.312, P=0.273	-

[§]Results from Pearson correlation tests (r, correlations coefficient) with age, symptom duration, MDS-UPDRS part III motor score as a measure of disease severity, motor fluctuation duration, MDS-UPDRS part IV as a quantitative measure of motor fluctuations, BDI and MoCA. Displayed are only significant correlations for clarity.

[#]Results from Mann-Whitney-U test. Displayed are only significant test results for clarity.

[$]Test results are from multivariate regression analyses with entering the candidate independent covariates age, sex, symptom duration, MDS-UPDRS part III motor score as a measure of disease severity, motor fluctuation duration, MDS-UPDRS part IV as a quantitative measure of motor fluctuations, BDI and MoCA as well as the corresponding variable of interest. Multicollinearity of candidate variables were excluded by Pearson correlation test (|r|<0.5). Results were confirmed by hierarchical multiple linear regression models. Non-significant *P* values of model coefficients (without Bonferroni correction; *P*<0.05) have been omitted for clarity, but significant results after adjusting *P* values for multiple testing are marked in bold letters (correlations: *P*<0.0014 for PKG quantitative scores and *P*<0.0021 for PKG daily times; regressions: *P*<0.013 for PKG® quantitative scores and *P*<0.016 for adPMD daily times).

Supplementary Table 4. Validity parameters of the adPMD for the detection of clinical observer Off ratings in balanced datasets.

	Accuracy (%)	Balanced accuracy (%)	Sensitivity, recall (%)	Specificity (%)	Precision (%)	Cohen's κ	MCC	F1-score
mBKS (standard cut-off)								
Original dataset	64%	63%	61%	65%	34%	0.21	0.22	0.44
Randomly undersampled balanced dataset	63%	63%	64%	62%	63%	0.26	0.26	0.63
Randomly oversampled balanced dataset	65%	65%	65%	65%	63%	0.30	0.30	0.64
Randomly combined under-/oversampled dataset	63%	63%	73%	54%	61%	0.27	0.27	0.69
mBKS (calibrated cut-off)								
Original dataset	72%	64%	51%	78%	42%	0.27	0.27	0.46
Randomly undersampled balanced dataset	66%	65%	53%	78%	70%	0.31	0.32	0.61
Randomly oversampled balanced dataset	65%	65%	50%	79%	70%	0.30	0.31	0.59
Randomly combined under-/oversampled dataset	63%	64%	63%	64%	69%	0.27	0.27	0.66
PD home diary								
Original dataset	26%	77%	63%	91%	71%	0.56	0.56	0.67
Randomly undersampled balanced dataset	79%	79%	63%	95%	92%	0.58	0.61	0.75
Randomly oversampled balanced dataset	78%	78%	65%	91%	88%	0.56	0.58	0.75
Randomly combined under-/oversampled dataset	78%	78%	65%	91%	88%	0.56	0.58	0.75

Supplementary Table 5. Validity parameters from the 2×2 contingency tables for the detection of clinical observer On ratings in balanced datasets.

	Accuracy (%)	Balanced accuracy (%)	Sensitivity, recall (%)	Specificity (%)	Precision (%)	Cohen's κ	MCC	F1-score
Standard cut-off								
Imbalanced test dataset	56%	52%	36%	69%	42%	0.05	0.05	0.39
Randomly undersampled balanced dataset	55%	55%	36%	74%	59%	0.11	0.12	0.45
Randomly oversampled balanced dataset	54%	54%	39%	69%	56%	0.09	0.46	0.08
Randomly combined under-/oversampled dataset	55%	55%	40%	69%	57%	0.09	0.10	0.47
Calibrated cut-off								
Imbalanced test dataset	61%	56%	39%	73%	46%	0.13	0.13	0.43
Randomly undersampled balanced dataset	60%	59%	39%	78%	62%	0.18	0.19	0.48
Randomly oversampled balanced dataset	56%	55%	37%	73%	54%	0.10	0.11	0.44
Randomly combined under-/oversampled dataset	55%	55%	40%	69%	55%	0.09	0.10	0.46
PD home diary								
Original dataset	65%	66%	70%	62%	54%	0.31	0.32	0.61
Randomly undersampled balanced dataset	68%	68%	70%	66%	67%	0.36	0.36	0.69
Randomly oversampled balanced dataset	68%	68%	73%	63%	66%	0.36	0.35	0.69
Randomly combined under-/oversampled dataset	67%	67%	74%	61%	65%	0.35	0.36	0.69

Supplementary Table 6. Validity parameters from the 2×2 contingency tables for the detection of observer Dyskinetic state ratings in balanced datasets.

	Accuracy (%)	Balanced accuracy (%)	Sensitivity, recall (%)	Specificity (%)	Precision (%)	Cohen's κ	MCC	F1-score
mDKS (standard cut-off)								
Imbalanced test dataset	72%	65%	40%	90%	68%	0.32	0.35	0.50
Randomly undersampled balanced dataset	65%	66%	40%	91%	83%	0.31	0.35	0.54
Randomly oversampled balanced dataset	61%	61%	36%	87%	74%	0.23	0.27	0.48
Randomly combined under-/oversampled dataset	63%	63%	34%	92%	82%	0.27	0.32	0.49
mDKS (calibrated cut-off)								
Imbalanced test dataset	70%	67%	55%	78%	60%	0.34	0.34	0.57
Randomly undersampled balanced dataset	65%	66%	55%	77%	72%	0.31	0.32	0.62
Randomly oversampled balanced dataset	54%	55%	39%	71%	61%	0.10	0.11	0.47
Randomly combined under-/oversampled dataset	59%	59%	47%	71%	62%	0.18	0.18	0.53
PD home diary								
Original dataset	73%	68%	49%	86%	66%	0.38	0.39	0.57
Randomly undersampled balanced dataset	67%	67%	49%	85%	76%	0.34	0.36	0.60
Randomly oversampled balanced dataset	68%	68%	49%	86%	79%	0.36	0.39	0.61
Randomly combined under-/oversampled dataset	68%	68%	49%	86%	76%	0.36	0.39	0.61

Supplementary Figure 1

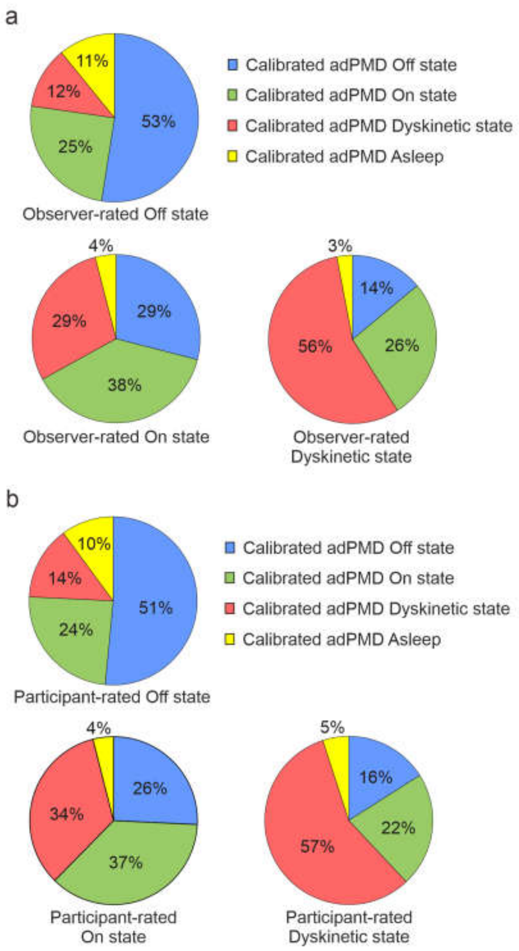

Supplementary Figure 1. Preferred choices in observer- and participant-documented diaries in the respective calibrated wearable accelerometer-based digital Parkinson's Motor Diary (adPMD) motor states.

Data based on 1,877 (70.9% of all time periods) simultaneous half-hourly calibrated adPMD and independent observer ratings serving as reference **(a)** and on 1,913 (72.3% of all time periods) simultaneous participant ratings of 63 participants **(b)**.

Supplementary Figure 2

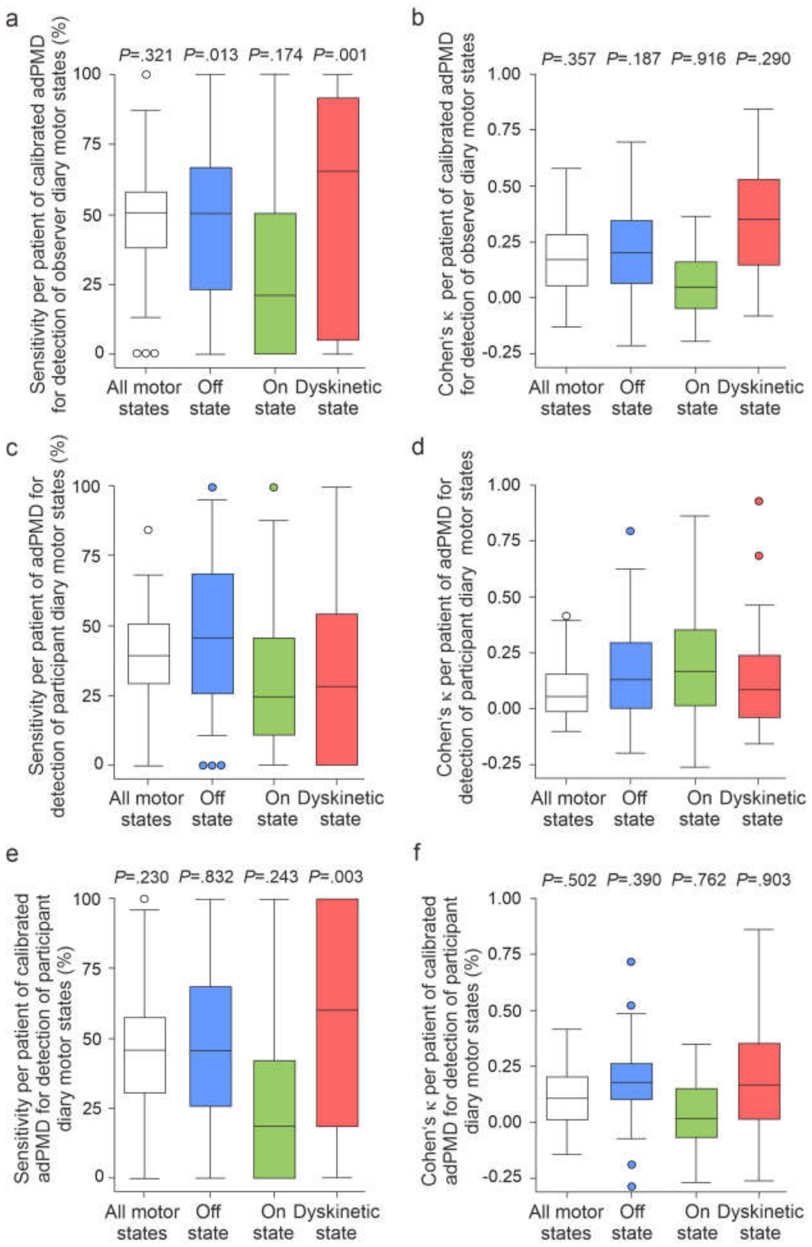

Supplementary Figure 2. Temporal agreement of observer- and participant-documented data and wearable accelerometer-based digital Parkinson's Motor Diary (adPMD) data on the participant level.

Sensitivities expressed in percent **(a)** and Cohen's κ values **(b)** of calibrated adPMD for the detection of all motor states (white colour), Off state (blue colour), On state (green colour) and Dyskinetic state (red colour) as rated by clinical observers and based on simultaneous half-hourly adPMD and clinical observer ratings in 63 participants. **(c-f)** Sensitivities in percent **(c,e)** and Cohen's κ values **(d,f)** of uncalibrated **(c,d)** and calibrated adPMD **(e,f)** for the detection of all motor states, Off state, On state and Dyskinetic state as rated by study participants in 63 participants. Boxplots are shown with a central mark at the median, bottom, and top edges of the

boxes at 25th and 75th percentiles, respectively, whiskers out to the most extreme points within 1.5 times the interquartile range, and outliers scoring more than 1.5×IQR but at most 3×IQR outside the quartiles. *P*-values are from Wilcoxon signed-rank tests when compared to uncalibrated adPMD motor diary data.

Supplementary Figure 3

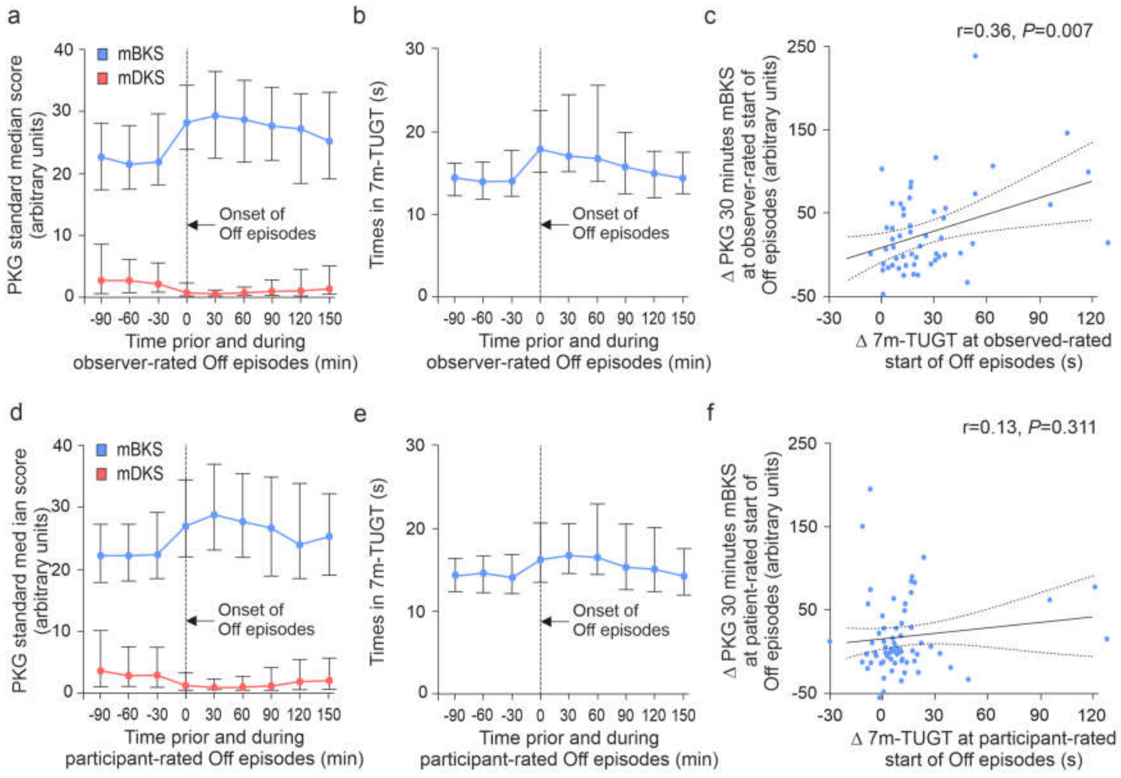

Supplementary Figure 3. Temporal agreement between PKG® scores, 7m-TUGT results and observer and participant-documented motor states over the time course of Off episodes.

(a,b,d,e) PKG quantitative scores (mBKS, mDKS) and 7m-TUGT test results synchronized to the onset of clinical observer-documented **(a,b)** and patient-rated **(d,e)** motor Off episodes. Off epsodes were defined as a minimum of 30 min Off preceded by at least 90 min with On time as judged by the clinical observers. **(c,f)** Correlation analyses of the changes in quantative bradykinesia scores from PKG assessment (mBKS) and 7m-TUGT at the start of Off **episodes** as documented by the observer **(c)** and the patient **(f)**. Solid line in represents the regression line with 95%CI (dotted lines). Numbers in right corner of the diagram represent Pearson correlation coefficient and *P*-values. Values are provided as medians ± interquartile ranges from 84 Off episodes in 51 participants for PKG data and 55 observed Off episodes in 32 participants for 7m-TUGT data for observer-rated diary data **(a-c)** and from 138 Off episodes in 56 participants for PKG data and 64 documented Off episodes in 32 participants for 7m-TUGT data for patient-documented data **(d-f)**.

Supplementary Figure 4

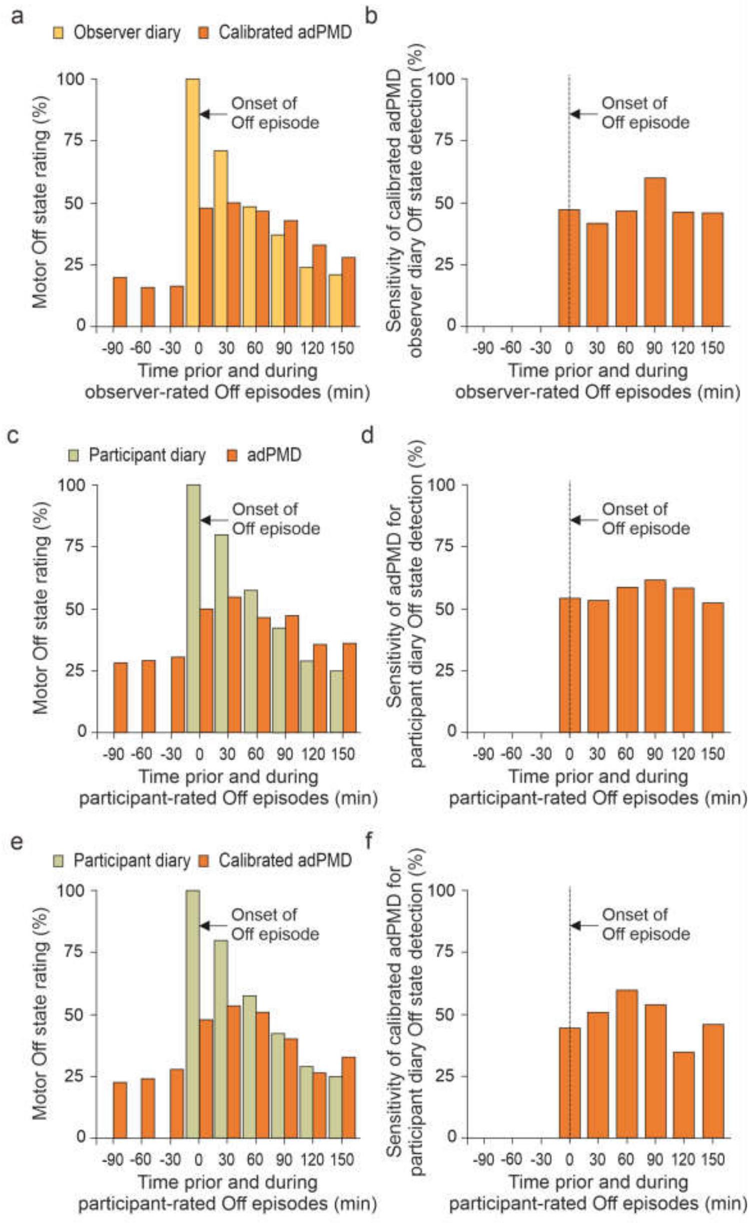

Supplementary Figure 4. Temporal agreement between observer and participant diaries and wearable accelerometer-based digital Parkinson's Motor Diary (adPMD) ratings over the time course of Off episodes.

(a) Proportions of Off responses on simultaneous observer ratings from 51 participants (yellow colour) and calibrated adPMD motor states (brown colour) synchronized to the onset of 115 patient-rated Off episodes, defined as a minimum of 30 min Off preceded by at least 90 min with On time as judged by the clinical observers. **(b)** Sensitivities of calibrated adPMD for the detection of observer-rated Off states synchronized to the onset of the observer-documented Off episodes. Corresponding Cohen's κ values ranged from -0.22 to 0.25. **(c-f)** Proportions of Off responses on simultaneous observer ratings from 51 participants (yellow colour) and uncalibrated adPMD **(c)** and calibrated adPMD **(e)** rated motor states (brown colour)

synchronized to the onset of 115 patient-rated Off episodes, defined as a minimum of 30 min Off preceded by at least 90 min with On time as judged by the clinical observers. Sensitivities of uncalibrated adPMD **(d)** and calibrated adPMD **(f)** for the detection of participant-rated Off states synchronized to the onset of the participant-documented Off episodes. Corresponding Cohen's κ values ranged from -0.13 to 0.29 for uncalibrated and from -0.22 to 0.25 for calibrated adPMD data. All values are provided as numbers.

Supplementary Figure 5

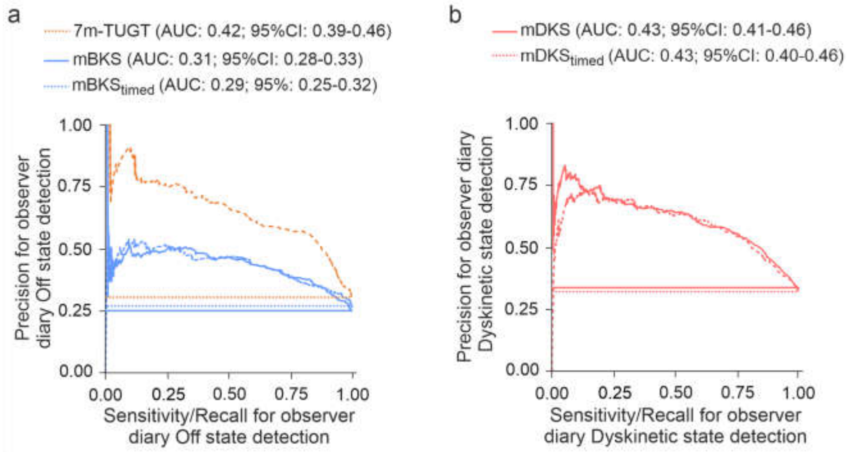

Supplementary Figure 5. Precision-recall curve (PRC) plot analyses of PKG® quantitative scores for the detection of participant-documented Off and Dyskinetic state.

(a,b) Precision-recall curve (PRC) analyses displaying the sensitivity (also recall) and precision of the 7m-TUGT results, mBKS and exactly timed mBKS recordings for diary classification with time frames of the 28 minutes (seven 2-minutes epoches before and 7 epoches after clinical rating) around the clinical ratings ($mBKS_{timed\ (28)}$) for the detection of observer-documented Off state **(a)** and mDKS and exactly timed mBKS recordings for diary classification with time frames of the 28 minutes (seven 2-minutes epoches before and 7 epoches after clinical rating) around the clinical ratings ($mDKS_{timed\ (28)}$) for the detection of observer-documented Dyskinetic state **(b)**. Data are based on 2,338 simultaneous half-hourly performed participant diary ratings, respectively with simultaneous PKG® recordings from 63 participants from 2 consecutive days (8 am to 6 pm). 7m-TUGT and timed PKG recordings were only performed in the German cohort (n=40, 1,361 half-hourly assessments).

Supplementary Figure 6

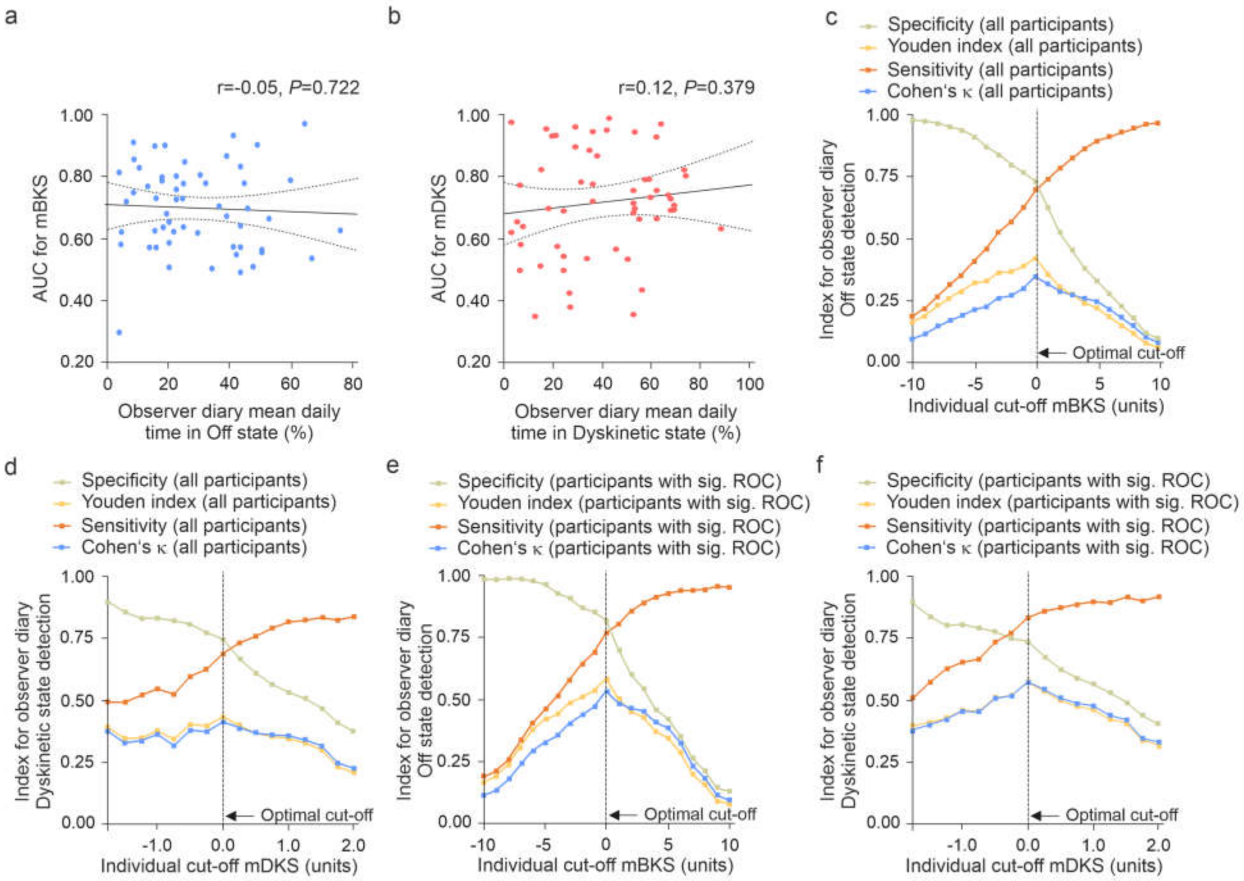

Supplementary Figure 6. Test performance of PKG® scores for the detection of observer-documented Off and Dyskinetic state using individualized cut-off values.

(a,b) Correlation analyses of AUC values from receiver operating curve (ROC) analyses of **(a)** mBKS for the detection of Off states as rated by the clinical observer and **(b)** mDKS for the detection of observer-documented Dyskinetic states compared with the respective percentage daily times in motor states. Numbers in right corner of scatter plot represent Pearson correlation coefficient and *P*-value. **(c-f)** Test performance indices (sensitivity, specificity. Youden index, Cohen's κ for the detection of observer-documented Off states by individual mBKS cut-offs **(c)** and Dyskinetic states by individual mDKS cut-offs **(d). (e,f)** Same test performance indices for the cohorts with significant discrimination of Off versus Non-Off states **(e)** and Dyskinetic versus Non-Dyskinetic states **(f)** as detemined by ROC analyses.

Supplementary Figure 7

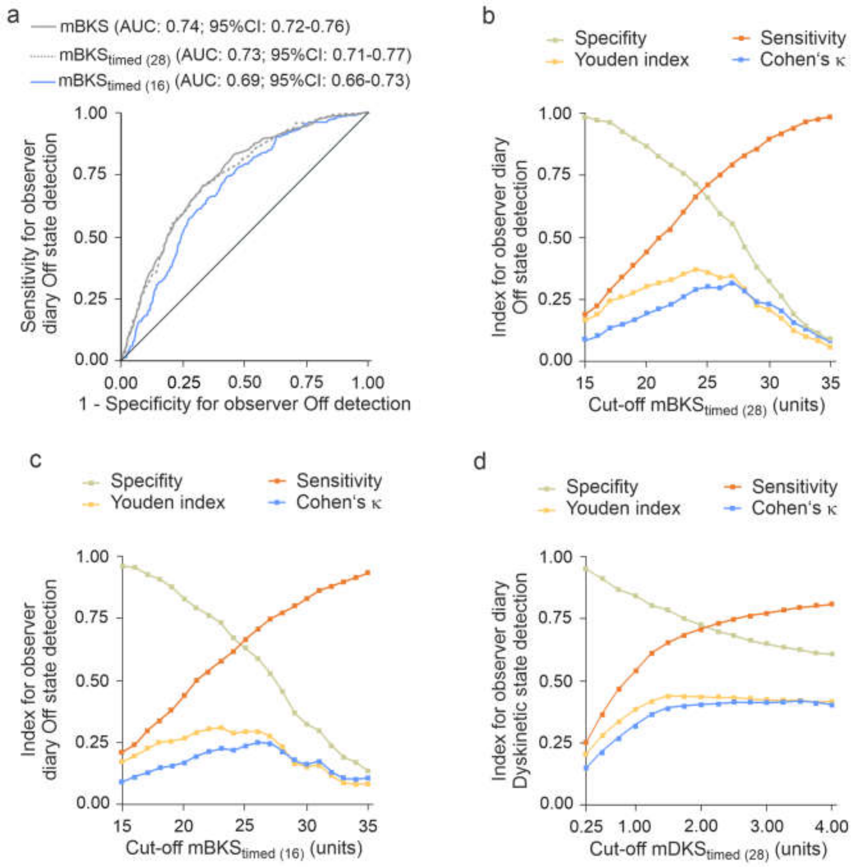

Supplementary Figure 7. Test performance of timed PKG® scores for the detection of observer-documented Off and Dyskinetic state.

(a) Receiver operating curve (ROC) analyses displaying the sensitivity and specificity of exactly timed mBKS recordings for diary classification with time frames of the 16 min (eight 2-min-epoches before rating) directly prior clinical rating (the ROC data of standard mBKS and timed $mBKS_{timed\ (28)}$ from ***Fig.*** *7* are displaed in grey for direct comparison). Test performance indices (sensitivity, specificity. Youden index, Cohen's κ for the dection of observer-documented Off state by timed $mBKS_{timed\ (28)}$ **(b)**, timed $mBKS_{timed\ (16)}$ **(c)** and for the detection of observer-documented Dyskinetic state by timed $mDKS_{timed\ (28)}$ **(d)**. Timed PKG® recordings were only performed in the German cohort (n=40, 1,361 half-hourly assessments).

Supplementary Figure 8

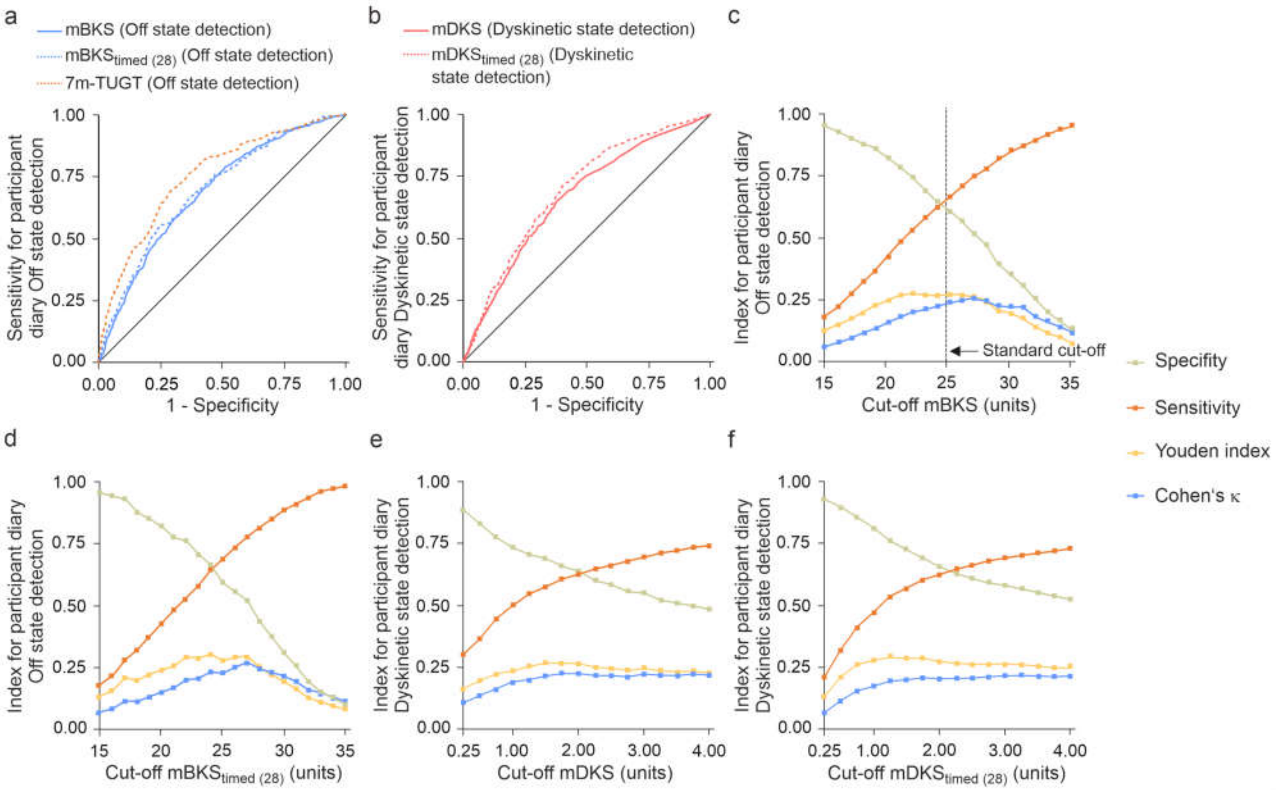

Supplementary Figure 8. Test performance of PKG® quantitative scores and 7m-TUGT results for the detection of participant-documented Off and Dyskinetic state.

(a,b) Receiver operating curve (ROC) analyses displaying the sensitivity and specificity of mBKS, $mBKS_{timed\ (28)}$ and 7m-TUGT for the detection of participant-documented Off state **(a)** and mDKS for the detection of participant-documented Dyskinetic state **(b)**. **(c,d)** Test performance indices (sensitivity, specificity. Youden index, Cohen's κ as displayed in **(c)**) for the dection of participant-documented Off state by mBKS **(c)** and $mBKS_{timed\ (28)}$ **(d)**. **(e,f)** Test performance indices (as displayed in **(c)**) for the dection of participant-documented Dyskinetic state by mDKS **(e)** and

$mDKS_{timed\ (28)}$ **(f)**. Data are based on 2,338 simultaneous half-hourly performed participant diary ratings, respectively with simultaneous PKG® recordings from 63 participants from 2 consecutive days (8 am to 6 pm). 7m-TUGT and timed PKG recordings were only performed in the German cohort (n=40, 1,361 half-hourly assessments).

Supplementary Figure 9

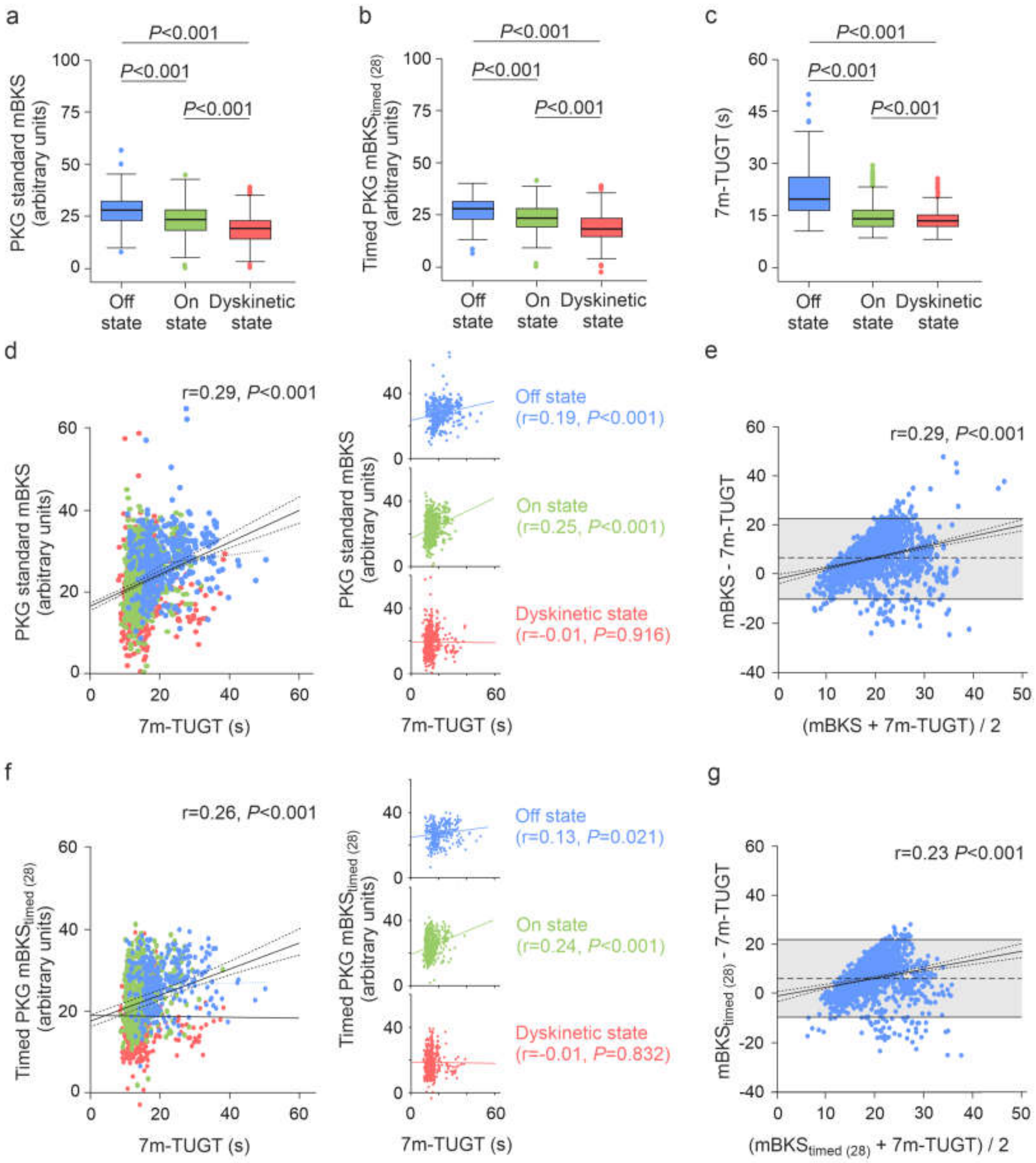

Supplementary Figure 9. Correlation of observer-rated diaries and 7 meter timed-up-and-go test data with PKG® bradykinesia scores (mBKS) on the half-hour time period level. Results of half-hourly detected quantitative PKG® mBKS or 7 meter timed-up-and-go test (7m-TUGT) data in 63 participants with respect to Off state, On state and Dyskinetic state as documented in the observer or the patient diary. **(a,b)** show the standard mBKS from PKG® recordings **(a)** and the timed 28 minutes around clinical rating mBKS ($mBKS_{timed\ (28)}$) **(b)** with respect to observer diary ratings. **(c)** 7m-TUGT results as a quantitative clinical bradykinesia rating with respect to observer diary entries. Boxplots are shown with a central mark at the median, bottom, and top edges of the boxes at 25th and 75th percentiles, respectively, whiskers out to the most extreme points within 1.5 times the interquartile range, and outliers scoring more than 1.5×IQR but at most 3×IQR outside the quartiles. Displayed *P*-values are from Kruskal-

Wallis tests with Dunn-Bonferroni *post-hoc* tests, corrected for multiple comparisons. **(d-g)** display the comparison of mBKS and 7m-TUGT results showing correlation analyses of standard mBKS **(d)** and timed mBKS from recordings exactly 28 minutes around clinical testing ($mBKS_{timed\ (28)}$) **(f)** and the corresponding Bland-Altman plots **(e,g)**. Insets on right side display the correlation of data clustered according to observer rated motor states. Solid lines represent the regression line with 95%CI (dotted lines) and the grey area in **(e,g)** the limit of argeement (mean±1.96 SD). Pointed lines represent the locally estimated scatterplot smoothing (LOESS) lines. Numbers in right corner of diagrams represent corresponding Pearson correlation coefficient and *P*-values. Data are based on 2,389 (90.3% of all time periods) simultaneous half-hourly performed observer diary ratings **(a)** with simultaneous PKG® recordings from 63 participants from 2 consecutive days (8 am to 6 pm). Similar half-hourly performed 7m-TUGTs and timed PKG® recordings **(b-g)** were only assessed in the German subcohort (n=40; 1,361 (81.0% of all time periods) simultaneous half-hourly assessments).

Supplementary Figure 10

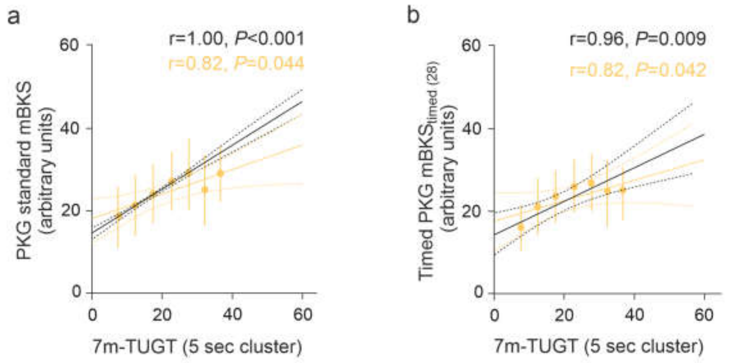

Supplementary Figure 10. Correlation of observer-rated diaries and 7 meter timed-up-and-go test data with clustered PKG® bradykinesia scores (mBKS) on the half-hour time period level.

Correlation of standard mBKS from PKG® recordings **(a)** and timed 28 minutes around clinical rating mBKS (mBKS$_{\text{timed (28)}}$) **(b)** seperated into 5 sec-TUGT clusters with respect to 7m-TUGT results. Beige circles and bars represent mean±SD mBKS values of 5 sec clusters of 7m-TUGT data. Beige solid lines represent the regression line with 95%CI (dotted lines) of all data clusters, and black solid lines represent the regression line with 95%CI (dotted lines) of data clusters <30 sec taking the ceiling effects of mBKS values at higher 7m-TUGT values into account. Numbers in right corner of diagrams represent corresponding Pearson correlation coefficient and *P*-values (beige: all data clusters; black: data clusters of data <30 sec). Data are based on 1,361 (81.0% of all time periods) simultaneous half-hourly assessments of the German subcohort (n=40).

Supplementary Figure 11

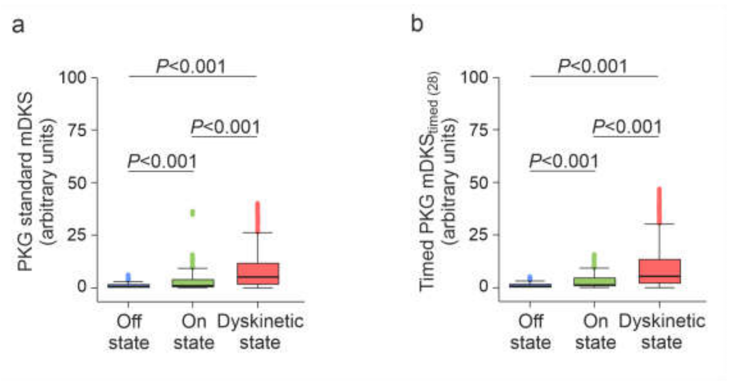

Supplementary Figure 11. Correlation of observer-rated diary data with PKG® dyskinesia scores (mDKS) on the half-hour time period level.

Results of half-hourly detected quantitative PKG® mDKS in 63 participants with respect to Off state, On state and Dyskinetic state as documented in the observer or the patient diary. **(a,b)** display the standard mDKS from PKG® recordings **(a)** and timed 28 minutes around clinical rating mDKS ($mDKS_{timed\ (28)}$) **(b)** with respect to observer diary ratings. Boxplots are shown with a central mark at the median, bottom, and top edges of the boxes at 25th and 75th percentiles, respectively, whiskers out to the most extreme points within 1.5 times the interquartile range, and outliers scoring more than 1.5×IQR but at most 3×IQR outside the quartiles. Displayed *P*-values are from Kruskal-Wallis tests with Dunn-Bonferroni *post-hoc* tests, corrected for multiple comparisons. Data are based on 2,389 (90.3% of all time periods) simultaneous half-hourly performed observer diary ratings **(a)** with simultaneous PKG® recordings from 63 participants from 2 consecutive days (8 am to 6 pm). Similar half-hourly performed timed PKG® recordings **(b)** were only assessed in the German subcohort (n=40; 1,361 (81.0% of all time periods) simultaneous half-hourly assessments).

Supplementary Figure 12

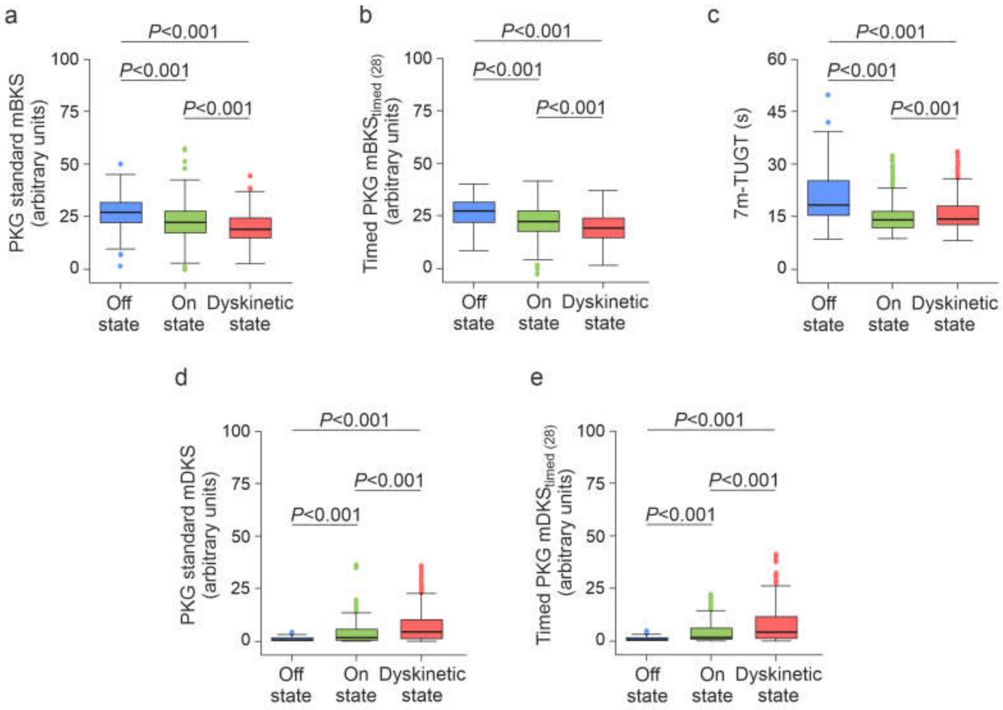

Supplementary Figure 12. Correlation of participant diaries and 7 meter timed-up-and-go test data with PKG® quantitative scores on the half-hour time period level.

Results of half-hourly detected quantitative PKG® or 7 meter timed-up-and-go test (7m-TUGT) data in 63 participants with respect to Off state, On state and Dyskinetic state as documented in participant diaries. **(a,b)** show the standard mBKS from PKG® recordings **(a)** and timed 28 minutes around clinical rating mBKS ($mBKS_{timed\ (28)}$) **(b)** with respect to participant diary ratings. **(c)** 7m-TUGT results as a quantitative clinical bradykinesia rating with respect to participant diary entries. **(d,e)** display the standard 30 minutes mDKS from PKG® recordings **(d)** and the timed 30 minutes around clinical rating mDKS **(e)** with respect to participant diary ratings. Boxplots are shown with a central mark at the median, bottom, and top edges of the boxes at 25th and 75th percentiles, respectively, whiskers out to the most extreme points within 1.5 times the interquartile range, and outliers scoring more than 1.5×IQR but at most 3×IQR outside the quartiles. Displayed *P*-values are from Kruskal-Wallis tests with Dunn-Bonferroni *post-hoc* tests, corrected for multiple comparisons. Data are based on 2,338 (88.4% of all time periods) simultaneous half-hourly performed participant diary ratings **(a,d)** with simultaneous PKG® recordings from 63 participants from 2 consecutive days (8 am to 6 pm). Similar half-hourly performed 7m-TUGTs and timed PKG® recordings **(b,c,e)** were only assessed in the German subcohort (n=40; 1,261 (81.0% of all time periods) simultaneous half-hourly assessments).
